# Evaluation of the effects of clearing agents, fixation, and process durations on cardiovascular tissue imaging with second harmonic generation and multi-photon modalities

**DOI:** 10.3389/fbioe.2025.1606425

**Published:** 2025-07-25

**Authors:** Maedeh Makki, Zachary A. Molander, Sergio A. Pineda-Castillo, Devin W. Laurence, Shubhra Singhal, Yasmin Eltwafsha, Gerhard A. Holzapfel, Tingting Gu, Chung-Hao Lee

**Affiliations:** ^1^ Biomechanics and Biomaterials Design Laboratory (BBDL), Department of Bioengineering, The University of California, Riverside (UCR), Riverside, CA, United States; ^2^ Department of Biomedical Engineering, University of Minnesota, Minneapolis, MN, United States; ^3^ Department of Anesthesiology and Critical Care, Children’s Hospital of Philadelphia (CHOP), Philadelphia, PA, United States; ^4^ Institute of Biomechanics, Graz University of Technology, Graz, Austria; ^5^ Department of Structural Engineering, Norwegian University of Science and Technology (NTNU), Trondheim, Norway; ^6^ School of Biological Sciences, The University of Oklahoma, Norman, OK, United States

**Keywords:** cardiovascular tissue, left anterior descending artery, second harmonic generation imaging, multi-photon imaging, benzyl alcohol benzyl benzoate (BABB) clearing, formalin fixation

## Abstract

**Introduction:**

Protocols for tissue clearing have been established and optimized for the central nervous system. However, significant modifications are required for clearing different tissue types. Therefore, effective optical clearing for cardiovascular tissue remains a major challenge. The goal of this study is to better understand the responses of porcine left anterior descending artery (LADA) to label-free multiphoton imaging.

**Methods:**

To this end, the effects of different clearing methods (i.e., benzyl alcohol benzyl benzoate–BABB and glycerol), formalin fixation, variations in formalin fixation times (0–240 min), and extended storage in BABB (up to 14 days) are investigated. We compare tissue characteristics under different conditions (e.g., tissue clearing reagent and/or tissue fixation), particularly with regard to tissue preservation and transparency across z-stacks (i.e., imaging depths).

**Results:**

The glycerol clearing method exhibited relatively lower tissue transparency, whereas BABB increased mean AF-AUC from 0.0035 ± 0.0009 to 0.1205 ± 0.0168 and SHG-AUC from 0.0003 ± 0.0002 to 0.0072 ± 0.0040 (*p*< 0.001), enabling robust signal intensities at deeper layers of LADA tissue. In addition, we observed that BABB preserves fluorescent signals even after extended tissue storage with no significant loss in integrity over 14 days. Finally, we found that formalin fixation in combination with the glycerol clearing method significantly improved tissue preservation compared to the glycerol clearing method alone. However, in combination with the BABB clearing method, fixation reduced tissue transparency and signal intensity compared to BABB clearing without fixation.

**Discussion:**

These findings establish BABB as the superior, label-free clearing agent for deep 3D multiphoton microscopy/second harmonic generation imaging of cardiovascular tissue and underscore the necessity of tailoring fixation parameters to the chosen clearing method.

## 1 Introduction

Significant progress in non-invasive imaging technologies have been made in recent decades, including the development of magnetic resonance imaging (MRI), computed tomography (CT), and positron emission tomography (PET) (Azaripour et al., 2016). These imaging techniques enable a resolution at the cellular level; however, specific research requires a subcellular resolution. In this context, the interface between established chemical nonlinear optical (NLO) techniques and biological microscopy has led to further advances in imaging capabilities, e.g., the development of multiphoton microscopy (MPM), second harmonic generation (SHG), and third harmonic generation (THG) imaging modalities (Campagnola, 2011).

Among these imaging modalities, MPM is a fast and non-invasive method for probing biological tissues at the subcellular level while maintaining a high resolution (Zipfel et al., 2003; Liang et al., 2017). MPM is known for its ability to generate label-free images using two-photon excited fluorescence (TPF) signals from intrinsic fluorophores, such as flavin adenine dinucleotide (FAD) and reduced nicotinamide adenine dinucleotide (NADH) (Croce and Bottiroli, 2014). This allows researchers to visualize large areas of unstained tissue while simultaneously providing real-time depth analysis. However, the accuracy of MPM imaging can sometimes be affected by sampling limitations (Chen et al., 2021).

On the other hand, SHG works by combining two photons of a lower energy to generate one with twice the frequency of the original light. In his 1986 pioneering work, Freund first used SHG for biological imaging, specifically to study collagen fibers in rat tail tendons (Freund et al., 1986). Due to the reliance on endogenous contrast, SHG microscopy offers several advantages for tissue imaging (e.g., direct visualization of tissue architecture without external dyes (Campagnola and Loew, 2003)). Technically, the SHG signal is generated by nonlinear interactions within the material via induced polarization, which significantly reduces photobleaching and phototoxicity compared to traditional fluorescence techniques. For SHG to occur, the environment must exhibit non-centrosymmetry (Zhuo et al., 2010) to an extent corresponding to the wavelength of the emitted light. This highlights the crucial role of molecular alignment within the medium. By using lasers in the near-infrared range (700–1000 nm), SHG microscopy can provide high-resolution images at depths of several hundred microns, enhancing its utility in biological research (Campagnola, 2011). Within the broad applications of SHG for imaging biological tissues, collagen- and myosin-based animal tissues are among the most extensively studied biological samples in SHG imaging (Chu et al., 2007; Lin et al., 2005; Plotnikov et al., 2006; Tiaho et al., 2007; Williams et al., 2005).

Despite these advances in imaging, light penetration depth is still insufficient to capture *complete* tissue images (Meador et al., 2020). Tissue opacity is mainly caused by the mismatch in refractive indices (RI) at the interfaces between the lipid and aqueous media (Chung and Deisseroth, 2013) and by the uneven distribution of scatterers such as lipids, collagen fibers and myofibrils (Richardson and Lichtman, 2015). The propagation of light through tissue results in attenuation due to absorption and scattering. This attenuation can be modeled by the Beer-Lambert law using (Equation 1)

$$I\left(z\right)=I_{0}e^{-\mu_{\text{eff}}z}$$ (1)

where $I_{0}$ is the initial intensity of the light, $I\left(z\right)$ is the light intensity at a depth *z*, and $\mu_{\text{eff}}$ is the effective coefficient accounting for both absorption and​ scattering (Plotnikov et al., 2006). An effective method to improve the penetration depth is to homogenize the RI mismatch of the biomolecule through tissue clearing (Costantini et al., 2019; Tuchin, 2005; Zhu et al., 2013). To achieve tissue transparency for imaging purposes while minimizing damage to the tissue, various chemical solutions have been developed, including benzyl alcohol benzyl benzoate (BABB), THF/DBE, Scale, SeeDB, CLARITY, 3DISCO, and CUBIC (Becker et al., 2012; Hama et al., 2011; Ke et al., 2013; Epp et al., 2015; Ertürk et al., 2012; Tainaka et al., 2014).

Overall, *ex vivo* tissue clearing methods can be divided into three main types based on their clearing principles and the chemical properties of the reagents used: *hydrophobic*, *hydrophilic*, and *hydrogel-based* methods (Ueda et al., 2020). Hydrophobic and hydrophilic clearing methods, also known as solvent-based and aqueous-based techniques, respectively (Zhu et al., 2021), each involve distinct processes with unique advantages and limitations (see Table 1). As discussed in several recent reviews (Richardson and Lichtman, 2015; Costantini et al., 2019; Ueda et al., 2020), the choice of the appropriate clearing agent depends on the specific biological sample and the experimental context. Therefore, no single agent is ideal for all situations. Most of the previously studied methods have been used for central nerve system (CNS) imaging (Yang et al., 2014). However, the research on clearing methods for cardiovascular tissues remains limited (Kolesová et al., 2016; Nehrhoff et al., 2016; Yokoyama et al., 2017), particularly the development of effective clearing protocols for cardiovascular tissue. Despite advances in tissue clearing and imaging, there are few studies on cardiovascular applications. For example, solvent-based approaches that include BABB were first used by Miller et al. (2005) and later refined by Kolesová et al. (2016) and Ivins et al. (2016). FluorClear BABB was introduced by Epah et al. (Epah et al., 2018). In addition, simple immersion techniques (e.g., FRUIT and MACS) have been employed for cardiac tissue by Xu H. et al. (2019); Xu J. et al. (2019) and Zhu et al. (2020).

**TABLE 1:** Summary of hydrophobic and hydrophilic clearing methods, techniques, and agents.

Clearing method	Process	Advantages	Common Agents/Techniques	Limitations
Hydrophobic clearing	Dehydrates tissue, dissolves lipids, and replaces water/lipids with high-refractive index (RI) organic solvents for transparency	High clearing efficiency, rapid RI matching	Dehydrating agents: ethanol, methanol, THF^1^ RI-matching agents: BABB, ECi^2^, DPE^3^, DBE^4^	Endogenous fluorescence may be compromised
Hydrophilic clearing (high-RI aqueous solutions)	Immerses tissue in aqueous solutions with a high RI to reduce RI mismatches	Better preservation of endogenous fluorescence	Agents: sucrose, fructose, glycerol, TDE^5^, formamide Techniques: FocusClear, SeeDB/SeeDB2	Less efficient clearing, limited effectiveness on whole organs
Hydrophilic clearing (hyperhydration and lipid removal)	Uses hyperhydration and lipid removal with water-soluble chemicals to enhance tissue transparency	Breaks down dense fibers, reduces overall RI	Agents: urea Technique: Scale	Incomplete transparency in large tissues

1 Tetrahydrofuran.

2 Ethyl Cinnamate.

3 Diphenyl Ether.

4 Dibenzyl Ether.

5 2,20-thiodiethanol.

Calle et al. (2014) combined BABB-based optical clearing with MPM and SHG to achieve subcellular, three-dimensional visualization of tissue-engineered constructs. Based on this pioneering work, Sung et al. (2016) evaluated and compared two optical clearing techniques—BABB (a representative of hydrophobic clearing) and glycerol (GLY, a representative of hydrophilic clearing)—for microstructural analysis of the left anterior descending artery. The previous method in literature minimized light scattering and enabled imaging at depths beyond 1 mm. Their method, which overcomes the spatial limitations of traditional 2D histology and allows researchers to capture intrinsic fluorescence and SHG signals, has been used to reveal the detailed microarchitecture (e.g., axially, helically, and circumferentially aligned collagen fibers in vascular grafts) and provide rapid, high-resolution volumetric data on decellularized and recellularized lung scaffolds. Building on this foundation, we propose in this work to utilize SHG and autofluorescence (AF) imaging to systematically develop an effective and feasible clearing approach for the microstructural analysis of cardiovascular tissue.

## 2 Materials and methods

### 2.1 The study groups

To thoroughly analyze the influence of various factors on optical tissue clearing processes on image transparency, we designed the following three studies: (i) Study 1 to examine the effects of different clearing methods (BABB, GLY, and noncleared–NC) and tissue fixation, resulting in a combination of six groups (unfixed: BABB, GLY, NC; fixed: BABB-F, GLY-F, NC-F); (ii) Study 2 to investigate the influence of fixation duration based on five study time points (0 – baseline from Study 1, 30, 60, 120, and 240 min of fixation in formalin); and (iii) Study 3 to evaluate potential changes in clearing due to long-term storage based on five study time points (0 – baseline, 1, 3, 7, and 14 days of storage in BABB). After Study 1, BABB was the only clearing reagent used in other studies. The objectives, conditions, and variables of these three studies are listed in Table 2.

**TABLE 2:** Summary of the proposed studies, including the study objectives, variables changed, and the experimental conditions or time points for tissue clearing and fixation.

Study	Objective	Variable changed	Experimental conditions or time points	Groups
Study 1	(i) Compare uncleared and cleared tissues	Clearing agent	BABB-cleared Glycerol-cleared uncleared	BABB, BABB-F, GLY, GLY-F NC, NC-F
	(ii) Compare unfixed and fixed tissues	Fixation	Fixation in formalin No fixation	
Study 2	Assess the effect of fixation time in formalin	Fixation time	0 (baseline-BABB), 30, 60, 120, and 240 min	F0, F30, F60, F120, F240
Study 3	Investigate the effect of varying clearing times within BABB	Clearing time	0 (baseline), 1, 3, 7, and 14 days	D0, D1, D3, D7, D14

### 2.2 Acquisition and preparation of LADA tissue

**FIGURE 1:**
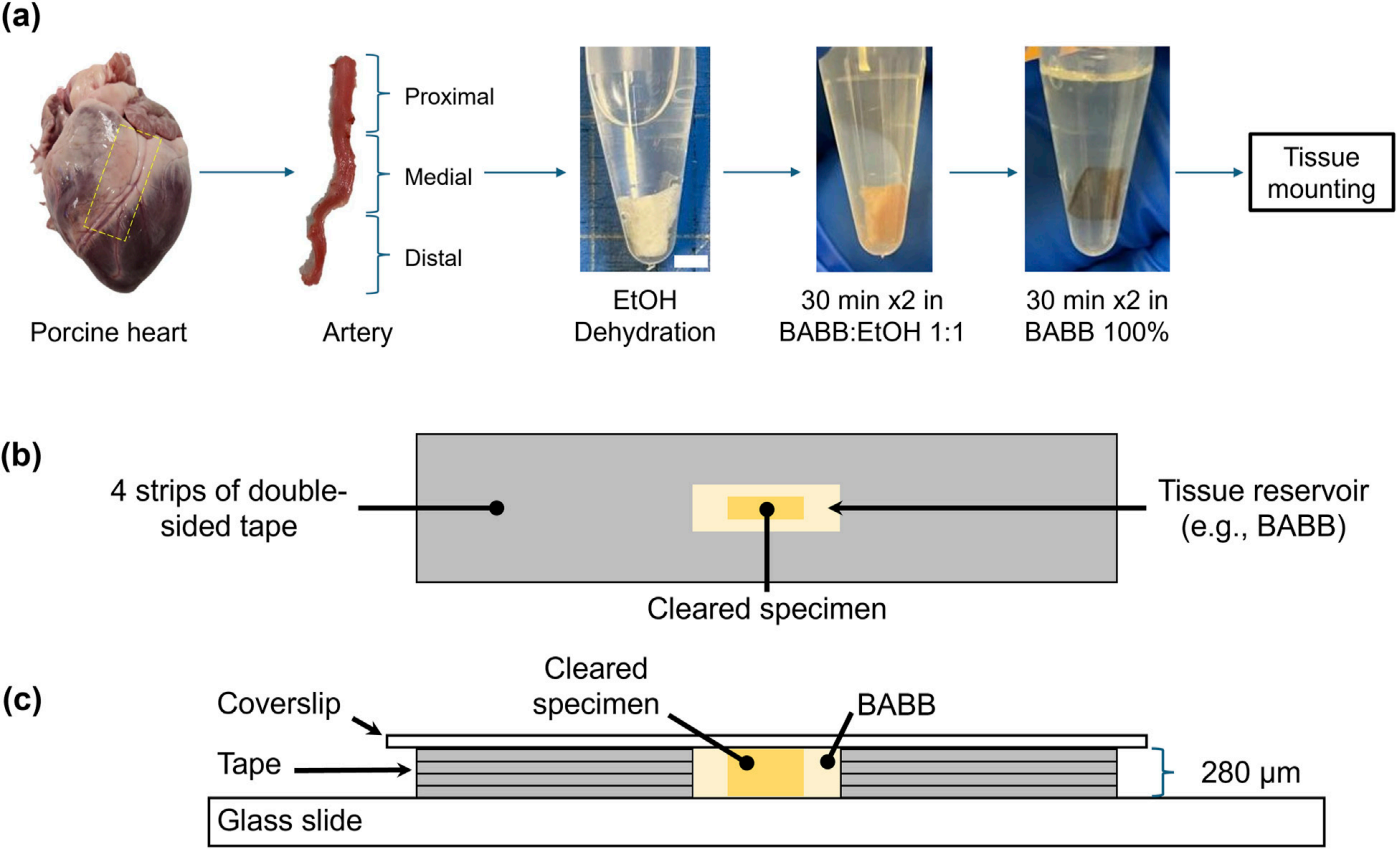
Schematic representation of the left anterior descending artery (LADA) clearing and mounting process: **(a)** acquisition of porcine LADA tissue and three tissue segments (proximal, medial, and distal); dehydration and clearing process of proximal LADA in various concentrations of ethanol and BABB (scale bar: 5 mm); **(b)** top view and **(c)** side views of the cleared specimens in a BABB reservoir with four layers of tape on a glass slide for imaging.

Porcine hearts (age 1–1.5 years and weight 80–140 Kg) were obtained from a USDA-approved source (Animal Technologies, Inc., Tyler, TX). Upon arrival at the lab, the left anterior descending artery (LADA) was isolated according to the previous protocol (Pineda-Castillo et al., 2022). Dissection began by taking sections from the anterior region of each heart, where the LADA is located (Figure 1a). The artery was then carefully separated as a single tissue specimen.

To fully expose the artery for subsequent analyses, the surrounding myocardium and connective tissues were carefully removed through longitudinal cuts, leaving only the arterial tissue. After isolation, the artery was segmented into three sections—proximal, medial, and distal—based on its relative distance from the aorta. This segmentation was carried out to ensure that imaging was focused on the largest region, specifically the *proximal* section (Pineda-Castillo et al., 2022). The LADA samples were then prepared for subsequent tissue optical clearing and microstructural quantification using multiphoton microscopy and SHG imaging. The tissue samples were temporarily preserved in a −20°C freezer unless immediately processed and tested.

### 2.3 Dehydration and clearing processes for the LADA tissue samples

To prepare for the clearing process, the tissues were first cut into strips. The crucial first step in preparing the tissues for clearing was dehydration. For this purpose, the tissues were immersed in ethanol-PBS solutions of increasing concentrations: 50%, 75%, 100%, and 100% (v/v), for 30 min each. Prior to dehydration, a rinse with phosphate-buffered saline (PBS) was necessary to remove any residual red pigment that would otherwise interfere with the clearing process. After dehydration, the tissues were stored in Falcon tubes (filled with 100% ethanol) until the day of MPM imaging (see Section 2.4).

Two different clearing agents were used for clearing: BABB and glycerol (GLY). The BABB solution was prepared as a 1:2 mixture of benzyl alcohol and benzyl benzoate (Dodt et al., 2007). The samples were subsequently cleared according to a previously described protocol (Calle et al., 2014). Briefly, the tissues were sequentially immersed in graded BABB (or GLY) solutions for 30 min each: a 1:1 mixture of ethanol and BABB (or GLY) followed by another 1:1 ethanol:BABB (or ethanol:GLY) solution, and 100% BABB (or GLY) twice, see Figure 1a.

### 2.4 Multi-photon microscopy (MPM) imaging

Once the tissues were optically transparent after clearing, they were mounted onto glass slides using double-sided tapes. This created an approximately 280-μm deep reservoir to hold both the tissue and the clearing agent (100% BABB or GLY) for subsequent multi-photon imaging (Figures 1b,c). Our primary goal here was to investigate the signal intensity of the arterial tissue at various depths using the *z*-stacks obtained from MPM imaging. Specifically, tissue autofluorescence (AF) and SHG signals were recorded using a Leica SP8 multiphoton system (Leica, Germany) equipped with a pulsed 80 MHz Ti:Sapphire laser (Chameleon, Coherent Inc., USA) with tunable wavelengths from 680 nm to 1080 nm.

An excitation wavelength of *λ*_ex_ = 830 nm with a pulse width of 10 fs was employed. The emission window was set to *λ*_em_ = 500–550 nm to detect the AF signals. For SHG imaging, the same excitation wavelength of *λ*_ex_ = 830 nm was used, but the emission was detected at *λ*_em_ = 410–420 nm. Both the AF and SHG signals were collected in z-stacks using a 20x NA:0.75 multi-immersion objective. The signals were subsequently detected using a hybrid detector (HyD) (Degiovanni et al., 2024).

### 2.5 Image analysis

After image acquisition, the resulting *z*-stack images were processed using ImageJ (National Institute of Health, Bethesda, MD) to extract signal intensity profile of the tissue. The goal of this step was to create.csv files for each experiment, tabulating *z*-depth data and the corresponding light intensity from multiple regions and specimens across different groups (Supplementary Table S1, Supplementary Material) of all three studies. The focus of the data was to evaluate differences in depth-discerned signal intensity between study groups defined in Section 2.1, which can provide insights into the structural and functional properties of the tissue.

### 2.6 Data analysis

The signal intensity profile of the tissue was subsequently processed and analyzed using an in-house MATLAB program (MathWorks, Natick, MA). The data processing involved normalizing the depths and intensities for each experiment relative to the maximum available imaging depth *z*_max_ and the maximum intensity (255), respectively.

To calculate the group-average normalized intensity over normalized depth for each group in each study, an in-house reproducing kernel (RK) algorithm in MATLAB was used to construct the approximate function for the intensity response and account for inconsistencies in tissue thickness between different regions and samples. This program first fitted the data from each *z*-stack using the RK shape functions, with their basis functions adjusted using a kernel function to ensure smoothness. The program then determined the normalized depth for each experiment incrementally at a fixed interval of 0.005 and computed the corresponding intensity from the RK-approximated intensity functions. Once the intensity reached a consistent set of normalized depth values, the group-average intensity was calculated for all experiments within each study. Further details on the RK function and the associated key equations can be found in Supplementary Appendix SA.

Additionally, we calculated the area under the curve (AUC) of each experiment by integrating the normalized intensity across the image depth. The AUC serves as a measure for a systematic comparative analysis of signal intensities across different groups. We then compiled the AUC values of all experiments in this study, sorted by group, specimen, region and AUC value, for the following statistical analyses/comparisons.

### 2.7 Statistical analysis

We reported the AUC results of all studies as the median with the first and third quartile values (Q1 and Q3). Data normality was tested using Q-Q plots and the Shapiro–Wilk test. For normally distributed data, comparisons between different tissue samples were performed using one-way or two-way ANOVA. For non-normal data, an aligned rank transform (ART) was performed. Differences were deemed statistically significant if *p* < 0.05 at α = 0.05. Note that the *p*-values for all statistically significant differences in AUC values between groups are provided in Supplementary Table S2, Supplementary Material. In addition, the *p*-values for AUC comparisons between BABB and BABB-F in different arterial layers (i.e., intima, media, adventitia) where statistically significant differences exist are shown in Supplementary Table S3, Supplementary Material. The *p*-values for the statistically significant comparison of tissue thickness between groups are provided in Supplementary Table S4, Supplementary Material. In addition, individual sample characteristics—e.g., tissue thickness (µm), AUC, maximum intensity, etc. are presented in Supplementary Tables S5, S7, S9 for the AF signal and Supplementary Tables S6, S8, S10 for the SHG signal (Supplementary Material). Supplementary Tables S5-S10 also include the median and interquartile range (IQR) for each characteristic within their respective group of Studies 1, 2, and 3.

## 3 Results

### 3.1 AF and SHG signals for study 1

**FIGURE 2:**
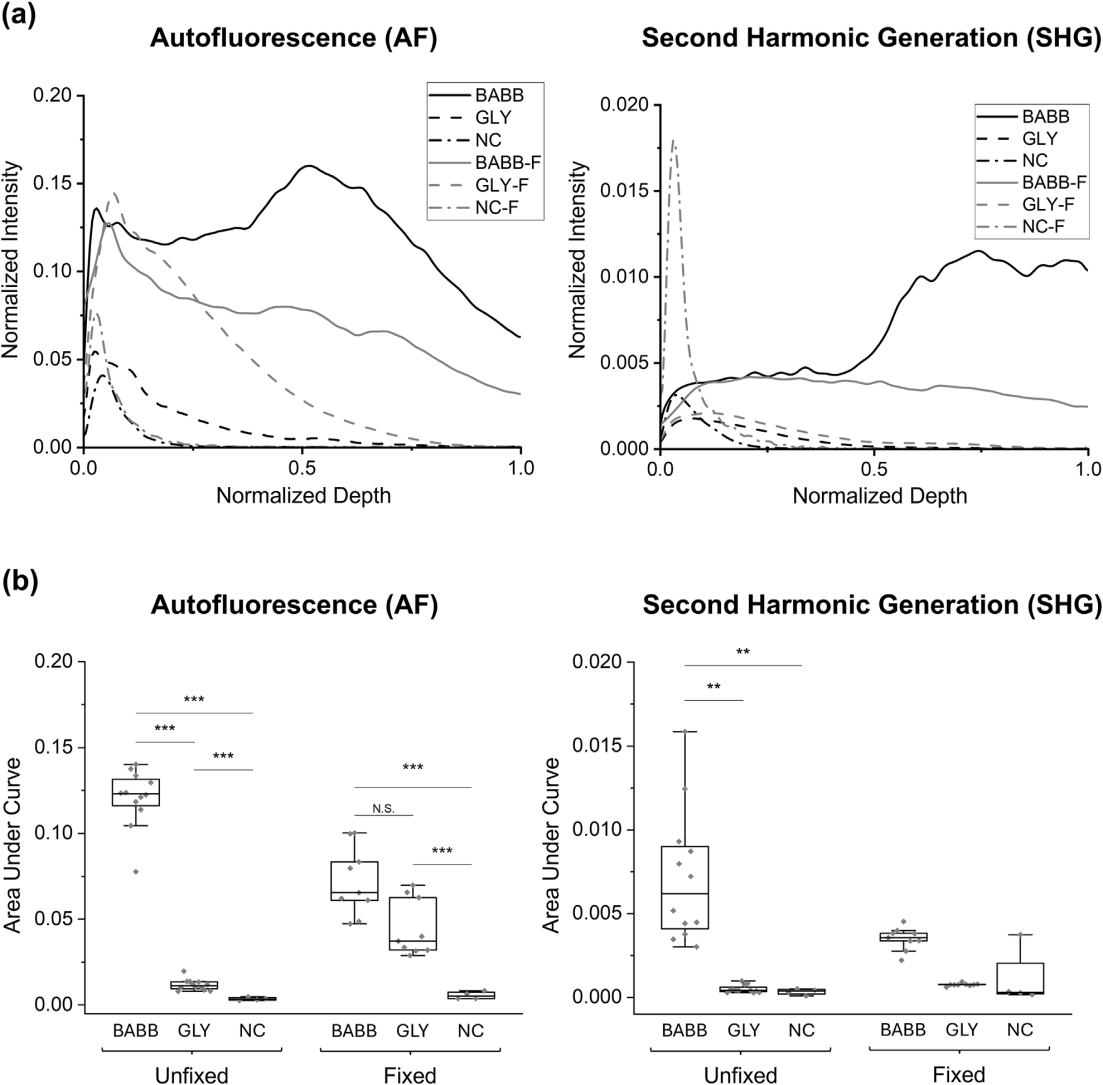
Comparison of **(a)** group-averaged normalized intensity curves and **(b)** the area under the normalized intensity curves for autofluorescence and SHG signals for Study 1. For clarity, significant comparisons between fixed and unfixed groups have been omitted. Significance levels: “N.S.” or not marked for *p* > 0.05, * for *p* < 0.05, ** for *p* < 0.01, *** for *p* < 0.001.

The group-average normalized intensities plotted against normalized depth revealed differences in signal detectability between the six groups for both AF and SHG imaging modalities (Figure 2a). Without clearing, i.e., in the NC (no clearing, no fixation) and NC-F (no clearing, with fixation) tissues, the signal rapidly diminishes and becomes undetectable in the near-surface layers (i.e., *z*/*z*_max_<0.2) for both AF and SHG signals. In contrast, glycerol-cleared tissues (i.e., GLY and GLY-F) exhibit a slower decay of the signal intensity, with the signal disappearing deeper in the tissue (*z*/*z*_max_≈0.6–0.8). In BABB-cleared tissue, both fixed (BABB-F) and unfixed (BABB), the signal intensity decreases continuously but remains detectable throughout the entire depth (Figure 2a). The gradual decrease in signal intensity across the depth suggests that clearing with BABB is particularly well-suited for imaging deep LADA tissue structures.

**FIGURE 3:**
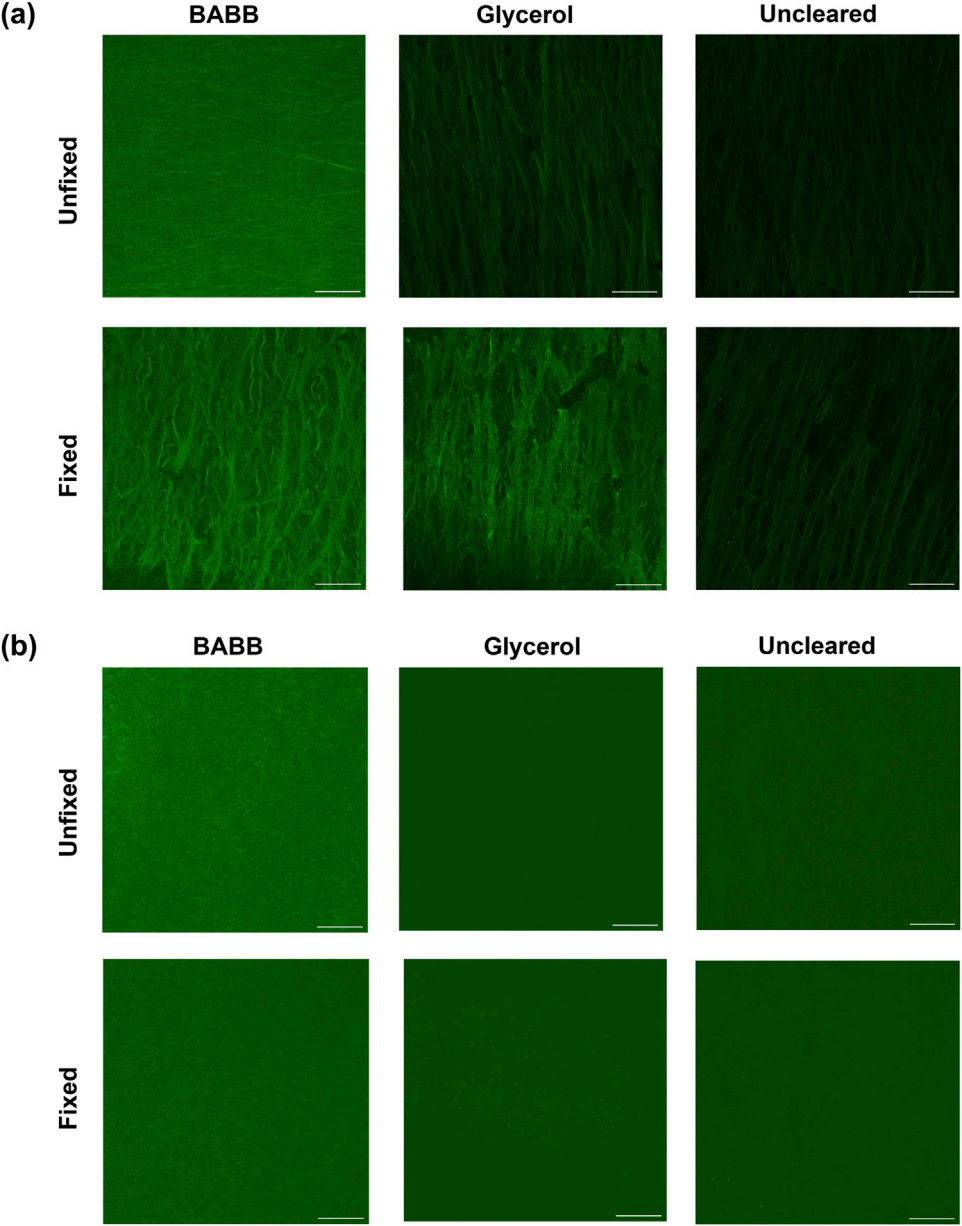
**(a)** Autofluorescence and **(b)** brightness and contrast-adjusted (Min: −39; Max: 119) SHG representative images show the pixel-wise maximum intensity for fixed and unfixed tissues cleared via BABB, GLY, or uncleared. Scale bars: 50 µm.

To better visualize the effects of tissue fixation and different clearing methods on signal retention, Figures 3a,b shows pixel-wise maximum intensity projections for AF and SHG in representative tissues: both fixed and unfixed, while cleared with BABB, glycerol, or uncleared. The intensities at discrete depths (i.e., every 20% of the total tissue thickness) in representative samples were compared to further assess the influence of these factors on AF and SHG signal retention as a function of imaging depth. These results are shown in Supplementary Material, Supplementary Figures S3,S4.

Regarding the AUC results for the AF signal, the smallest mean AUC was observed in the NC samples (0.0035 ± 0.0009), followed by the NC-F (0.0056 ± 0.0022), GLY (0.0117 ± 0.0032), and GLY-F (0.0446 ± 0.0165) samples. The highest areas were observed in the BABB-F (0.0720 ± 0.0199) and BABB (0.1205 ± 0.0168) samples, with the BABB group exhibiting the highest value (Figure 2b). Statistical analysis using the non-parametric ART test, based on the non-normality distribution of the data, reveals that both the clearing factor and its interaction with tissue fixation significantly influence the AUC (*p* < 0.001). However, fixation alone does not significantly influence the results (*p* = 0.059). A detailed pairwise analysis of the interaction between the clearing and fixation factors for the AF signal reveals statistically significant differences between all groups in this study (*p* < 0.05), except for the BABB-F and GLY-F pair (0.0720 ± 0.0199 vs. 0.0446 ± 0.0165, *p* = 0.102, Figure 2b).

On the other hand, the SHG analysis revealed the lowest AUC in the uncleared samples (0.0003 ± 0.0002), with slightly higher values in the GLY (0.0005 ± 0.0002), GLY-F (0.0008 ± 0.0001), NC-F (0.0011 ± 0.0017), and BABB-F (0.0035 ± 0.0007) samples. Moreover, consistent with the AF results, the highest AUC values were detected in samples cleared with BABB (0.0072 ± 0.0040, Figure 2b). Statistical analysis showed that tissue clearing significantly influences AUC (*p* < 0.001). Additionally, the interaction between clearing and fixation also has a statistically significant impact on the AUC (*p* = 0.008). These findings are consistent with the results obtained from the AF imaging. Although tissue fixation appears to have a minimal effect on the SHG signals, its influence is subtle and approaches the significance level (*p* = 0.056, α = 0.05). Due to statistically significant differences for the BABB vs. NC pair (0.0072 ± 0.0040 vs. 0.0003 ± 0.0002, *p* = 0.001), BABB vs. NC-F pair (0.0072 ± 0.0040 vs. 0.0011 ± 0.0017, *p* = 0.003), BABB-F vs. NC pair (0.0035 ± 0.0007 vs. 0.0003 ± 0.0002, *p* = 0.03), and BABB vs. GLY pair (0.0072 ± 0.0040 vs. 0.0005 ± 0.0002, *p* = 0.005), it is concluded that BABB enhances SHG signals compared to GLY and NC, while no significant difference is observed between GLY and NC (*p* > 0.05; Figure 2b).

Since BABB and BABB-F were identified as the more effective clearing agents, we carried out a comparative analysis on different coronary artery layers (i.e., intima, media, and adventitia), see Supplementary Figure S1, Supplementary Material. Our analysis revealed that fixed and unfixed tissues did not differ significantly in the intima and media layers (all pairwise comparisons yielded *p* > 0.1 with both AF and SHG imaging methods). However, in the adventitia layer, the AUC values were significantly lower for the BABB-F tissues than those for the BABB tissues (*p* < 0.05 for AF and *p* < 0.01 for SHG signals).

### 3.2 AF and SHG signals for study 2

**FIGURE 4:**
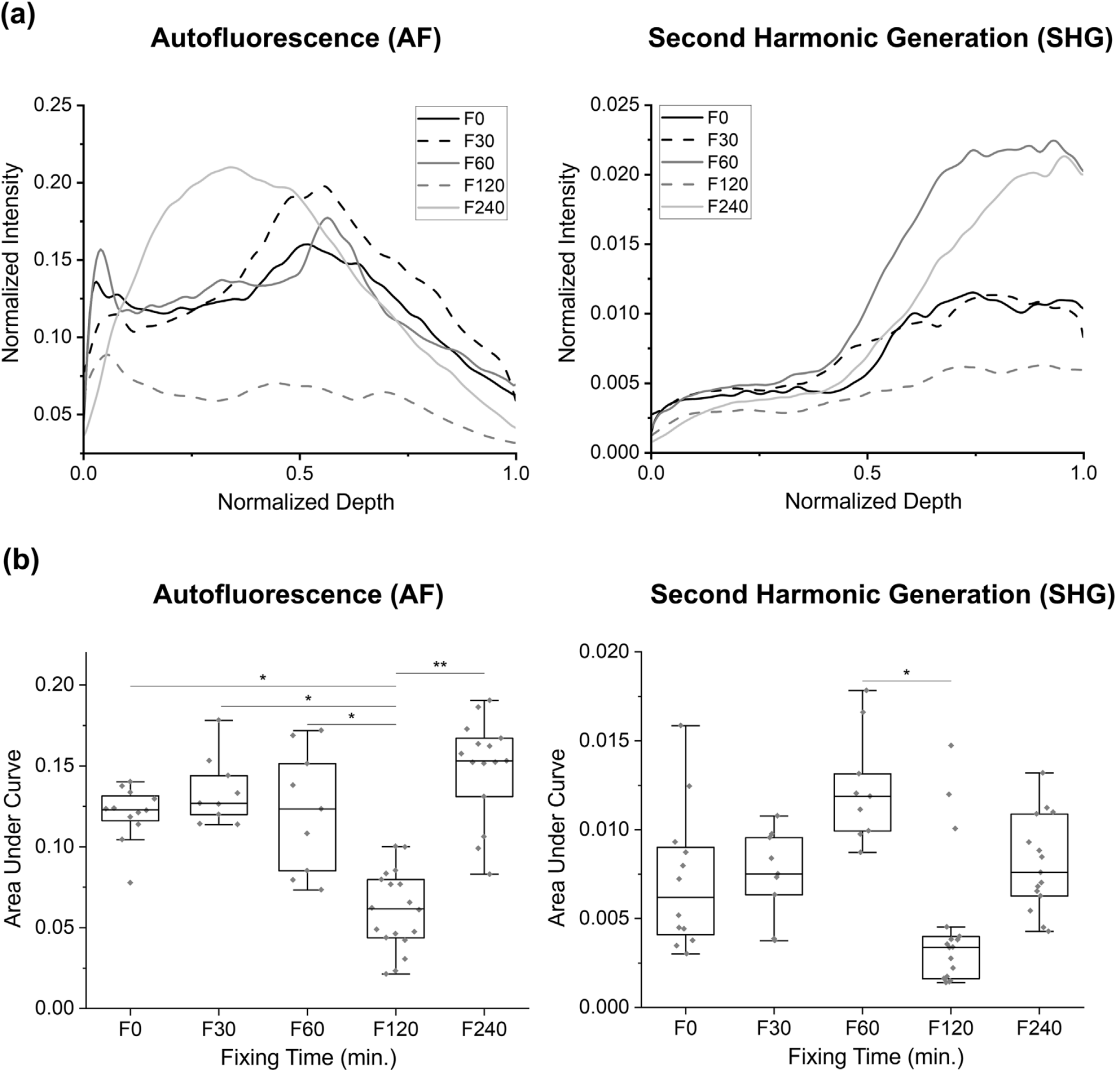
Comparison of autofluorescence and SHG intensity changes at different fixation times in Study 2: observations of intensity change at various fixing times in the BABB clearing: **(a)** the group-averaged normalized intensity curves, and **(b)** the area under the normalized intensity curves for each sample. Only significant values are displayed: * for *p* < 0.05, ** for *p* < 0.01.

In this study, the groups are based on the time the tissue was stored in the fixation solution, with durations of 30, 60, 120, and 240 min, as well as a 0-min fixation baseline from the unfixed BABB in Study 1. When examining the AUC values in these groups, a noticeable reduction in the mean AUC value was observed for the F120 group in the AF modality. Statistical analysis using one-way ANOVA after a normality test revealed a significant reduction in AUC for the F120 group compared to the F0, F30, F60, and F240 groups for the AF signals (0.0608 ± 0.0244 vs. 0.1205 ± 0.0168, 0.1344 ± 0.0210 , 0.1222 ± 0.0380, and 0.1496 ± 0.0290 , *p* < 0.031, Figure 4b). In SHG imaging, no statistically significant differences were observed between the different groups (*p* > 0.1 for all pairwise comparisons), except for the comparison between F120 and F60, where a tendency towards significance was observed (0.0043 ± 0.0039 vs. 0.0123 ± 0.0031, *p* = 0.048). Further, no significant differences were found between the F0, F30, F60, and F240 groups for either imaging modality (*p* > 0.05).

### 3.3 AF and SHG signals for study 3

**FIGURE 5:**
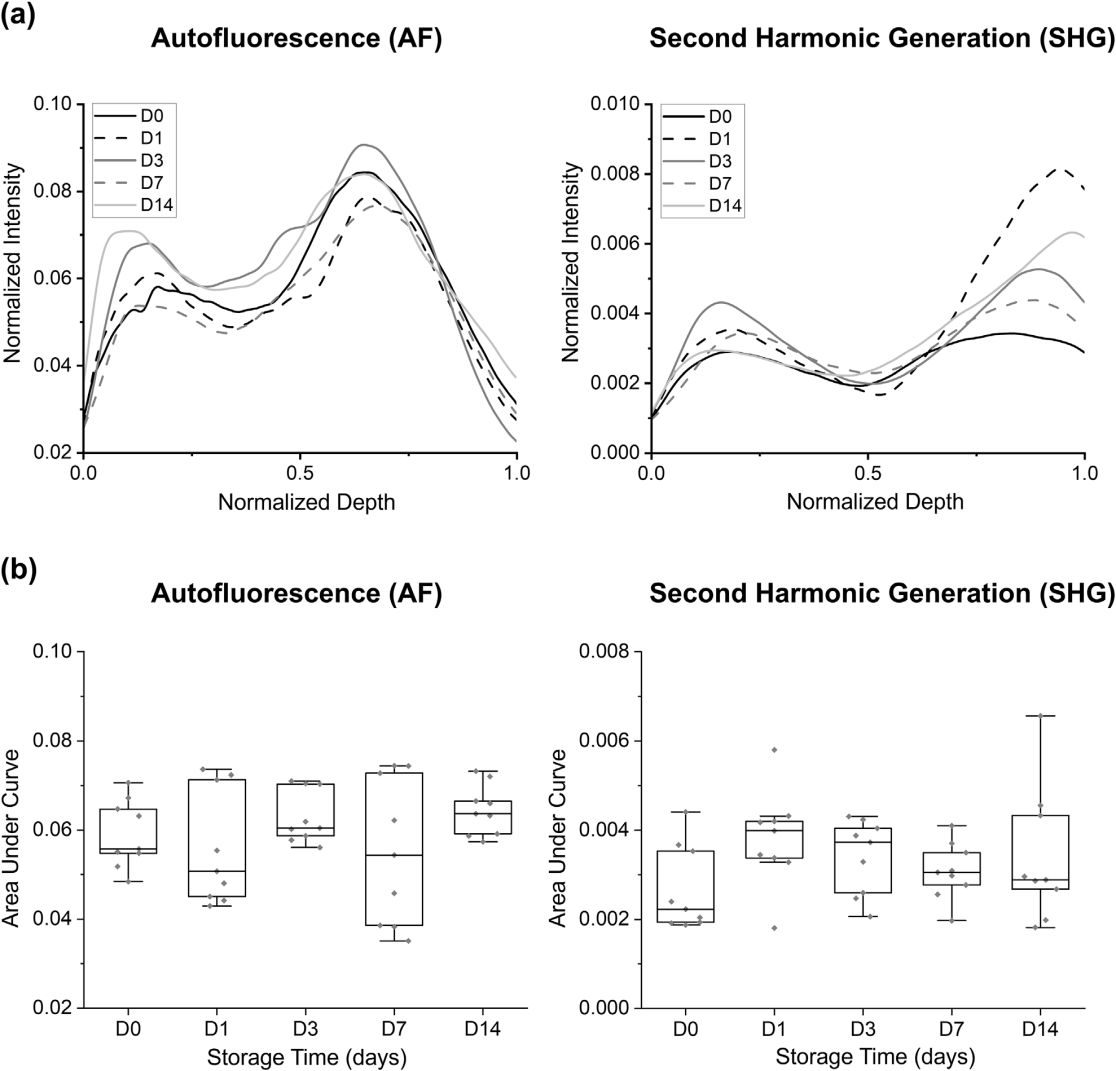
Comparison of autofluorescence and SHG intensity changes at different storage times in BABB: **(a)** group-averaged normalized intensity curves and **(b)** area under the normalized intensity curves for each sample. No significant differences were observed/displayed.

This study evaluated the influence of extended tissue storage in BABB (0, 1, 3, 7, and 14 days) on the AF and SHG signal intensities (Figure 5). Both the AF and SHG signals exhibited fluctuations over the course of extended BABB-storage: i.e., the AF signal showed the highest AUC at D14 (0.0644 ± 0.0056) and the lowest at D7 (0.0551 ± 0.0164), whereas the SHG signal reached its minimum intensity at D0 (0.0027 ± 0.0009) and its maximum at D1 (0.0038 ± 0.0011). Despite these fluctuated patterns, statistical analyses revealed no significant differences (*p* > 0.05) in AUC values across the storage durations for either AF or SHG. This suggests that longer storage in the BABB does not substantially affect the signal intensity.

Remark: While the AUC results did not indicate significant differences between groups, statistically significant differences (*p* < 0.05) in tissue thickness were observed—particularly among the (D1, D7), (D1, D14), (D3, D7), and (D0, D7) groups. These differences should be considered when interpreting the findings of this study (see Supplementary Figure S2, Supplementary Material).

## 4 Discussion

Optical tissue clearing is a widely recognized technique that enables quantitative 3D imaging in advanced microscopy by making tissue transparent for detailed imaging. The success of tissue clearing techniques depends not only on the chemical properties of the clearing agents but also on factors such as lipid content, structural components (e.g., extracellular matrix), and tissue fixation (Lee et al., 2016; Seo et al., 2016). However, the clearing of cardiovascular tissue—e.g., the left anterior descending artery, the focus of this study—poses greater challenges than the clearing of tissues such as the brain. Cardiac tissue presents a significant challenge for fluorescent labeling techniques such as immunolabeling and immunohistochemistry due to its dense, fibrous, and muscular structure. This complexity not only complicates tissue clearing in thicker or mature hearts but also limits the diffusion of antibodies, which are critical for effective labeling. Moreover, the high autofluorescence of intrinsic proteins, such as myoglobin and sarcomeric proteins, can obscure specific signals and interfere with fluorescent dyes, often requiring spectral shifting to distinguish true signals from background noise. The extensive vascular network further complicates the process, as residual blood and hemoglobin absorb light and create additional background interference. Moreover, the preservation of fluorescent proteins poses a significant challenge, e.g., BABB-based clearing methods can degrade fluorescence signals within a few days, complicating subsequent analysis (Kolesová et al., 2016). These factors, including poor fluorescent protein preservation, restricted antibody diffusion, and high autofluorescence, have contributed to the paucity of studies in the literature on the clearing method for cardiovascular tissue.

To address this knowledge gap, the present study carefully optimized the clearing protocols for cardiovascular tissue without the use of immunolabeling. The two commonly used clearing reagents—BABB and glycerol—were compared, along with fixation conditions and clearing durations. Both AF and SHG imaging methods evaluated tissue characteristics and quantified image transparency by measuring signal intensities after each protocol. For the tissue clearing method, BABB, a hydrophobic clearing agent (Lu et al., 2021), was chosen due to its suitability for connective tissue, ease of use, and lack of specialized equipment (Azaripour et al., 2016). However, glycerol was also tested as a representative hydrophilic clearing method (Jiang et al., 2008).

Study 1 shows that BABB provided significantly higher AUC results for both AF and SHG signals than glycerol. Glycerol-cleared tissues also showed increased AF signal intensity compared to uncleared tissues; however, no significant SHG signal intensity was observed between GLY and NC. We therefore assume that glycerol cannot outperform BABB in clearing because it relies on a simple immersion method. In particular, the AF signals observed in the emission window (500–550 nm) are most likely attributed to elastin in the LADA tissue (Zoumi et al., 2004). Elastin exhibits strong intrinsic fluorescence due to its unique cross-linked structure (Samouillan et al., 2002). In contrast, collagen, although a less prominent AF source in cardiovascular tissues, is a robust generator of SHG signals due to its highly ordered triple-helical structure (Zoumi et al., 2004; Du et al., 2025), typically detected in the range of 410–420 nm. BABB enhances elastin fluorescence by several mechanisms. First, it removes lipids, which both scatter light and quench fluorescence. Second, its high RI (∼1.55) (Becker et al., 2012) closely matches that of elastin (RI ≈ 1.51) (Costantini et al., 2019; Jacques, 2013), thereby reducing optical scattering and improving image depth (Fujimoto et al., 2000). Third, benzyl alcohol may partially denature proteins (e.g., tryptophan, tyrosine, and cross-linked desmosine) and expose buried fluorescent residuals, thus increasing the fluorescence quantum yield (Thirumangalathu et al., 2006; Singh et al., 2010). In comparison, glycerol improves elastin fluorescence, but to a lower extent. This lower efficacy is likely due to its inability to remove lipids and its lower RI (∼1.44) (Costantini et al., 2019), resulting in less effective optical clearing compared to clearing with BABB (Richardson and Lichtman, 2015). Achieving the necessary RI with highly concentrated glycerol solutions presents a significant challenge due to their high viscosity, which directly influences the diffusion coefficient of the clearing agents. Further, other challenges include precipitation at room temperature and the entrapment of air bubbles in the solution (Richardson and Lichtman, 2015). It has been found that reducing the viscosity of highly viscous fluids, such as glycerol (Azaripour et al., 2016), can improve the penetration of reagents (Yu et al., 2024).

Since our study compared only one representative of each of the solvent- and aqueous-based clearing methods, we compared BABB with alternative clearing agents available in the literature. Compared with Scale (Hama et al., 2011) and CUBIC (Susaki et al., 2014), both of which employ high-concentration urea, BABB delivers a clear advantage in terms of speed and transparency: Scale, for example, requires weeks to months to reach complete transparency, whereas CUBIC typically takes ∼1–2 weeks, and both cause substantial tissue swelling, which distorts microanatomical details (Hama et al., 2011; Susaki et al., 2014). Although CLARITY (Chung et al., 2013) excels at preserving endogenous fluorescence and enabling deep immunolabeling through hydrogel embedding and lipid removal, the original electrophoresis protocol requires 1–2 days of active clearing and specialized equipment, making it less ideal for high-throughput workflows. Although BABB causes moderate isotropic shrinkage, this uniform size reduction allows for the acquisition of 2–3 times larger volumes within the optimal imaging field of the light sheet without sacrificing subcellular resolution (Pan et al., 2016). Collectively, these comparisons suggest that BABB remains the clearing agent of choice when speed, depth, and controlled dimensional changes are critical.

In Study 2, despite some variation in AUC with formalin fixation duration, SHG imaging revealed no statistically significant differences across fixation times, except between the 60- and 120-min groups. In AF imaging, only the 120-min fixation resulted in a significantly lower AUC than all other durations, while no other pairwise comparisons reached statistical significance. Formalin, which contains formaldehyde, is a widely accepted protein fixative (Fox et al., 1985). Formalin-fixation induces covalent cross-links between proteins as well as between proteins and nucleic acids through hydroxymethylene bridges (French and Edsall, 1945; Helander, 1994). Werner et al. (2000) have suggested that such cross-links can mask epitopes by perturbing the tertiary structure of proteins. One possible explanation for the observed AUC reduction after 120 min is that prolonged fixation may *temporarily* alter the local chemical environment of harmonophores and fluorophores. This reduces their accessibility or alters their conformation, resulting in reduced signal intensity. The lower AUC observed after 120 min could also be due to biological variability: i.e., an intrinsically low autofluorescence signal in the tissue group examined at this timepoint. The latter explanation is supported by the lack of a similar reduction in the 240-min group. However, the exact mechanism is still unclear and is beyond the scope of this work.

In Study 3, we found that the long-term storage of cleared samples in BABB did not cause significant difference in the AUC values—as a measure of signal intensity—during the extended storage in this solution. This suggests that the clearing process is relatively fast, consistent with other reported results where BABB was used in skin or gingiva samples cleared within only 1–4 h (Azaripour et al., 2016). Furthermore, in this study, AUC values did not deteriorate significantly after long-term storage in BABB of up to 14 days (D14) compared to the first day (D0). This suggests that BABB effectively preserves fluorescent structures in LADA tissues, allowing fluorescence signals to remain detectable even after prolonged storage (∼2 weeks). This observation regarding the preservation of fluorescent proteins is consistent with previous findings from studies on skin and gingiva samples (Azaripour et al., 2016), as well as other studies using different protocols (e.g., FluoClearBABB (Schwarz et al., 2015), uDISCO (Pan et al., 2016), and vDISCO (Cai et al., 2019)), all of which used BABB as an RI-matching agent and concluded that tissue remains stable during storage over long periods of time.

## 5 Study limitations and future work

Although our study highlights promising outcomes, such as enhanced imaging depth and a relatively fast, minimally invasive clearing protocol, some limitations of this work must also be acknowledged. First, only one representative of each solvent-based and aqueous-based clearing method was selected, and no comparative analysis of the different clearing agents within each method was performed. Second, although the effects of sequential tissue clearing in the four graded solutions (combinations of BABB and ethanol) were evaluated using imaging and intensity data analysis, this pilot comparative study (see Supplementary Appendix SB) was limited to a single region from four distinct specimens. Due to the small sample size, no statistical analysis was performed for this pilot comparative study. Third, we observed a pronounced decrease in autofluorescence after 120 min of formalin fixation and attributed this decrease to two possible hypotheses that require further validation. Fourth, imaging was limited to small strips of the proximal portion of the LADA tissue, which may have influenced the results due to the limited tissue volume. Although two-photon microscopy offers greater penetration depth and is less affected by scattering than confocal microscopy, it has inherent limitations, including the ability to image only one single plane at a time and to capture large volumes of tissue simultaneously (Costa et al., 2019). Therefore, the findings of this study and their implications should be interpreted with caution, particularly given the potential bias due to the limited imaging area. Finally, in this study we utilized endogenous autofluorescence and second harmonic generation to enhance sensitivity to native fluorophores and collagen. This allowed us to bypass the usual blocking and antibody incubation steps. However, these steps remain important considerations for adapting the protocol to broader histochemical assay applications.

To further refine the comparison between aqueous-based and solvent-based clearing methods, future studies should evaluate additional aqueous-based approaches. One promising direction is the investigation of hyperhydration-based clearing (see Table 1). One promising direction in tissue clearing is the use of hyperhydration-based methods (see Table 1). These techniques reduce the tissue’s refractive index (RI) primarily through urea-induced protein dispersion and increased hydration, rather than relying solely on matching the RI with a high-index clearing solution. The Scale technique (Hama et al., 2011), for example, follows this principle by combining urea-driven protein denaturation with hydration in a glycerol-based medium. Urea disrupts hydrogen bonding within proteins, leading to partial denaturation and separation of dense, high-RI protein complexes, thereby lowering the overall RI. Glycerol serves to modulate tissue expansion, mitigate excessive hydration, and preserve structural integrity by reducing fragility (Richardson and Lichtman, 2015).

Additional experiments could be performed for each clearing step of this protocol to enable a more detailed statistical analysis of the different clearing stages. The significant loss of autofluorescence after 2 hours of formalin fixation might also represent an uncharacterized phenomenon that deserves targeted explorations at the molecular level. Future work could also focus on optical clearing analysis of the entire artery or vein in the cardiovascular system to validate the results for tissues with larger volumes. Future studies should also address histochemical analysis in BABB-cleared tissue, considering specific optimal protocols to improve staining quality. For example, extended PBS washes are recommended for effective removal of BABB residuals (Zhao et al., 2020), while detergents combined with prolonged incubation can enhance tissue permeability (Zhao et al., 2020). Antibody penetration and stability may be improved by extended incubations (Pomaville and Wright, 2023), the use of Fab fragments or nanobodies (Ariel, 2017; Li et al., 2015), and neutralization of the pH during rehydration. To preserve the fluorescence signal and minimize the background signal, researchers can further consider the use of solvent-resistant fluorophores such as Alexa Fluor (Panchuk-Voloshina et al., 1999), increased Tween-20 concentrations, and bovine serum albumin (BSA) in antibody buffers (Piña et al., 2022).

## 6 Conclusion

In this work, we conducted a comparative study to evaluate the efficacy of *ex vivo* clearing techniques on representative cardiovascular tissue (LADA) using AF and SHG imaging. We investigated the clearing agents, BABB and glycerol, and assessed their effect with and without formalin fixation, and compared the results with those on uncleared tissue. We further observed that both BABB and glycerol significantly enhance the AF signals compared to NC tissues, with BABB additionally characterized by a significant increase in SHG intensity compared to glycerol-cleared and NC tissues. Formalin fixation showed significant alterations in the AF signal (i.e., a decrease in AUC with BABB and an increase with glycerol), but no notable changes in SHG signals. We also conducted studies on fixation duration and prolonged tissue storage in BABB. The results showed no significant difference in the signal intensity of the AF or SHG signals between 30, 60, and 240 min of fixation. However, a fixation after 120 min resulted in a significantly lower AF signal compared to all other durations and a lower SHG signal compared to a fixation of 60 min. Finally, the suitability of the BABB agent for LADA tissue was demonstrated by its ability to bind fluorescence molecules even after extended storage in the clearing solution.

## Data Availability

The datasets generated and analyzed for this study (i.e., all the AF and SHG imaging.LIF files) can be found in the Zenodo repository (https://doi.org/10.5281/zenodo.15633822).
