# Supplementary material for: Evaluation of the effects of clearing agents, fixation, and process durations on cardiovascular tissue imaging with second harmonic generation and multi-photon modalities
Source: Front Bioeng Biotechnol. 2025 Jul 25;13:1606425. doi: 10.3389/fbioe.2025.1606425 (PMC12331724; doi:10.3389/fbioe.2025.1606425)
Supplement: Supplementary file 1 [file Supplementaryfile1.docx]

Supplementary Material

# Overview

This Supplementary Material section provides supplementary tables and figures for the manuscript “*Evaluation of the effects of clearing agents, fixation, and process durations on cardiovascular tissue imaging with second harmonic generation and multi-photon modalities*,” authored by Maedeh Makki, Zachary A. Molander, Sergio A. Pineda-Castillo, Devin W. Laurence, Shubhra Singhal, Yasmin Eltwafsha, Gerhard A. Holzapfel, Tingting Gu, Chung-Hao Lee.

In this section, the comparison of the area under the normalized intensity curves of the arterial layers (intima, media, adventitia) for BABB and BABB-F from Study 1 and the comparison of the sample thicknesses among the test groups in each study are shown in **Supplementary Figure 1** and **Supplementary Figure 2**, respectively. **Supplementary Figure 3 & 4** present the representative images throughout the tissue to visually contrast the clearing techniques in Study 1.

In addition, **Supplementary Table 1** contains the summary of specimen and region distribution across studies. **Supplementary Table 2** shows the *p*-values for comparisons between groups in each of the three studies (see Table 1 in the main text). **Supplementary Table 3** lists the *p*-values for comparisons between BABB, BABB-F in the arterial layers of Study 1. **Supplementary Table 4** summarizes the *p*-values for the comparison of sample thicknesses among different groups in each study. **Supplementary Tables 5-12** show individual sample characteristics.

# Supplementary Figures


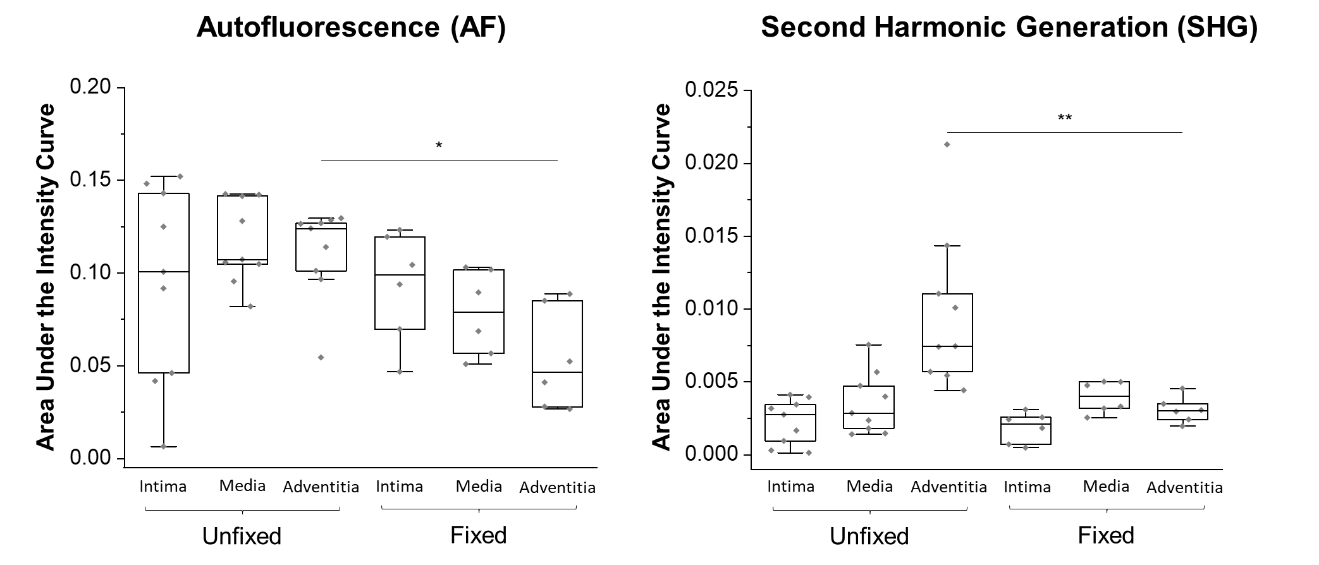


**Supplementary Figure 1.** Comparison of the area under the normalized intensity curves for autofluorescence (AF) and second harmonic generation (SHG) signals of different arterial layers (i.e., intima, media, adventitia) for BABB and BABB-F from Study 1. Note that comparison between layer types in different tissue fixing techniques were not included. (Significance levels: * for *p*<0.05,
** for *p*<0.01)


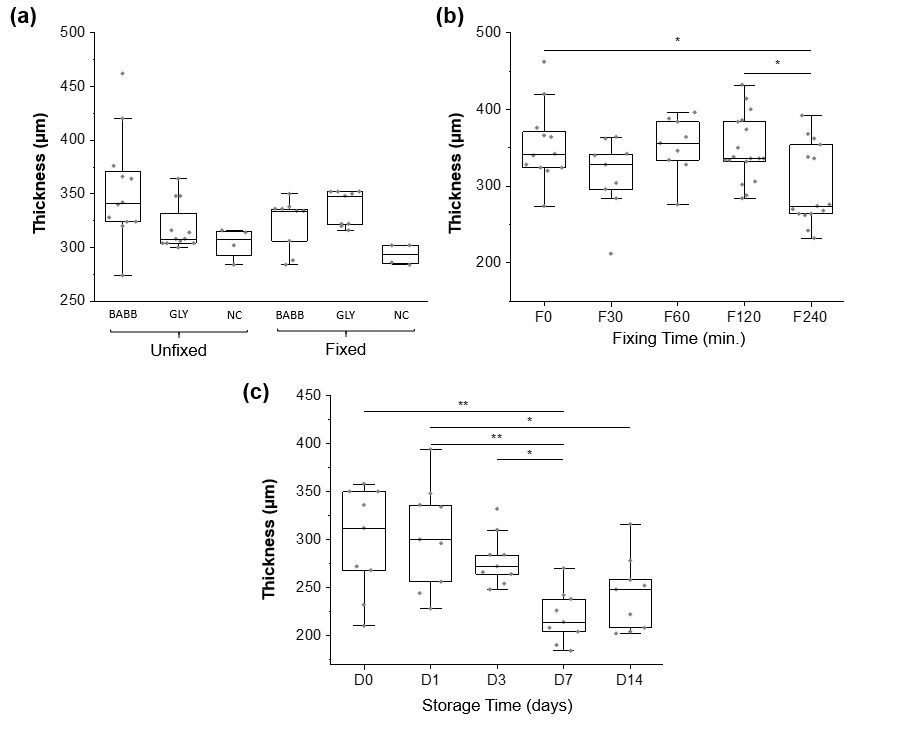


**Supplementary Figure 2.** Comparison of sample thickness in each of the three studies: (a) Study 1 for different clearing methods, (b) Study 2 for different formalin fixation times, and (c) Study 3 for different BABB storage times. (Significance levels: * for *p*<0.05, ** for *p*<0.01)


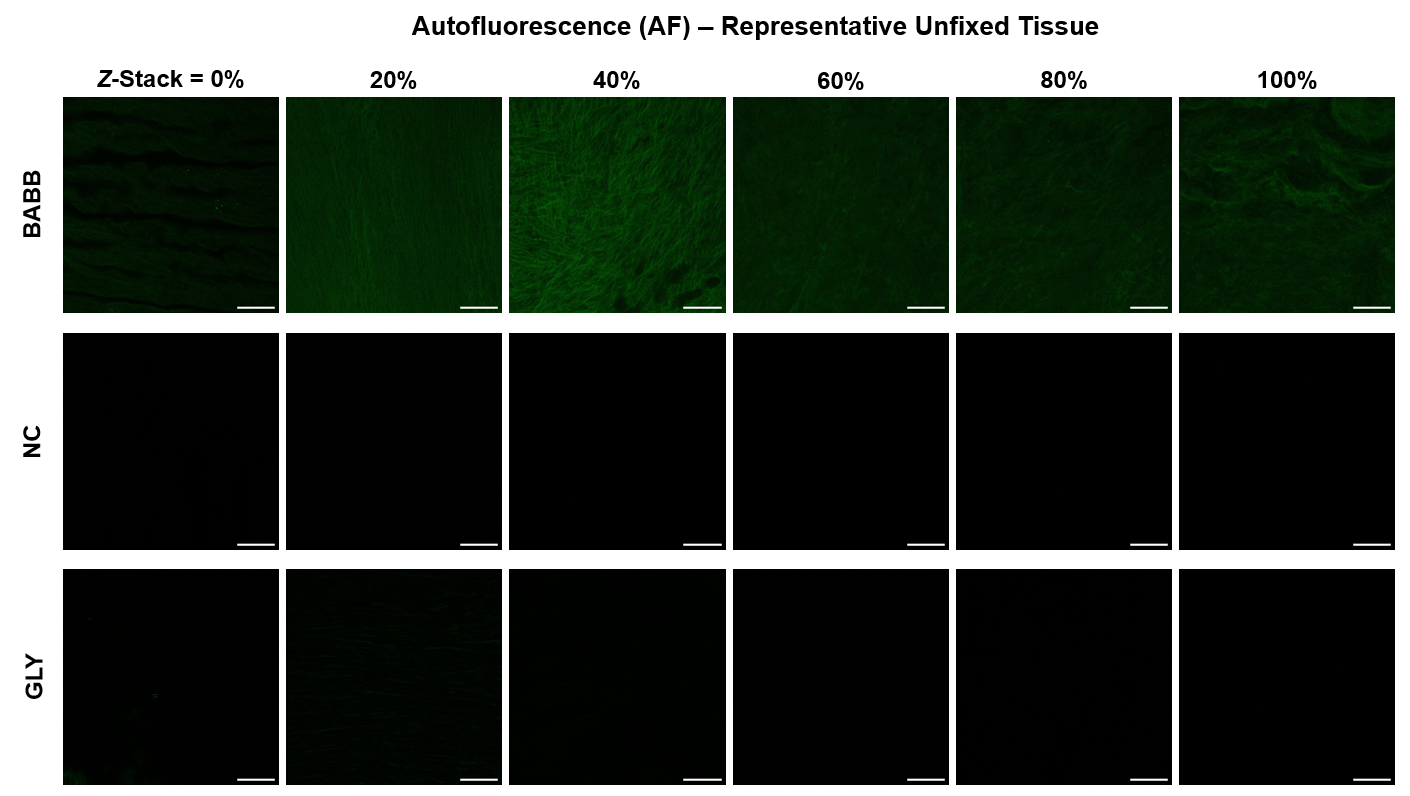


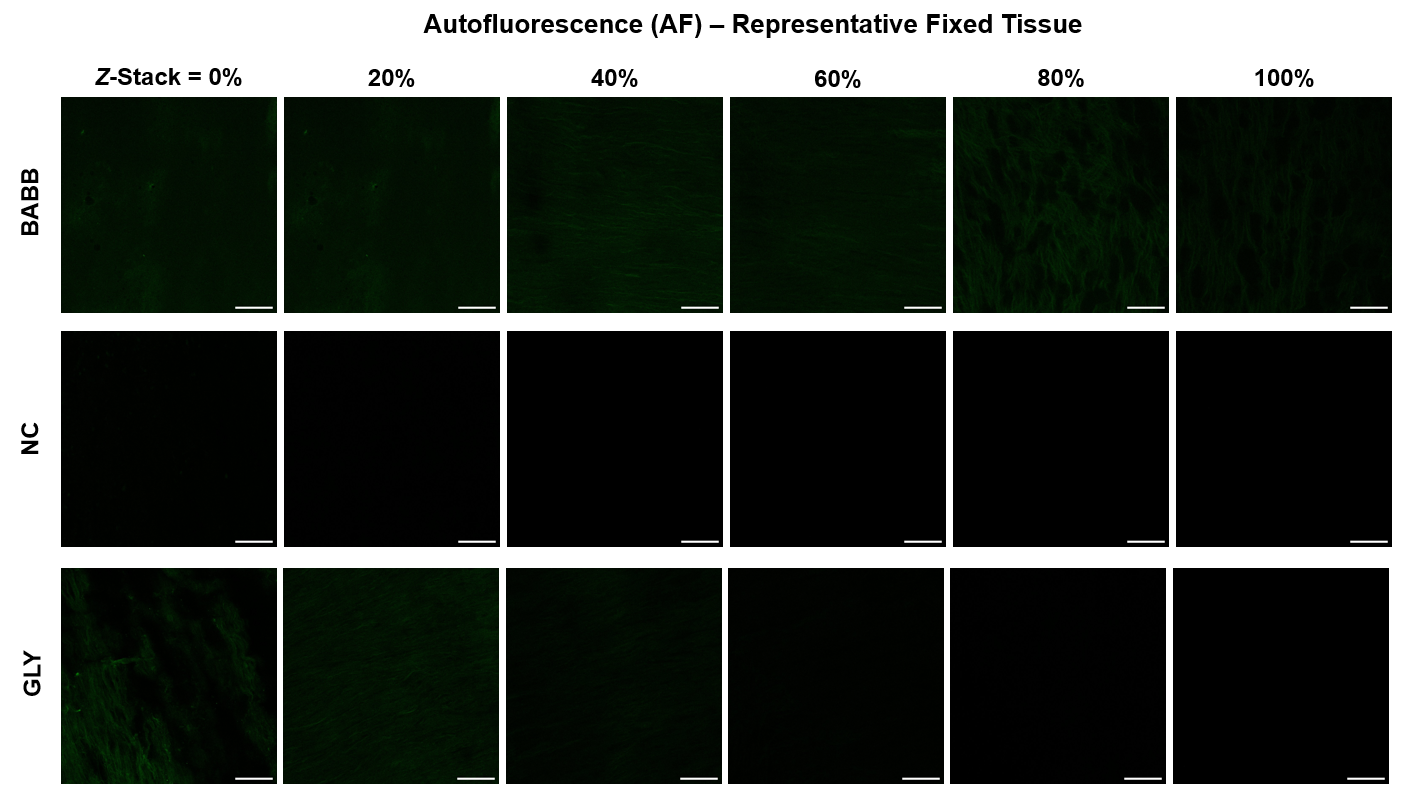


**Supplementary Figure 3.** Representative microscopic images of the autofluorescence signal vs. imaging depth for (*top row*) unfixed tissues and (*bottom row*) fixed tissues, cleared via BABB, glycerol (GLY), or uncleared (NC). Scale bars=50 µm


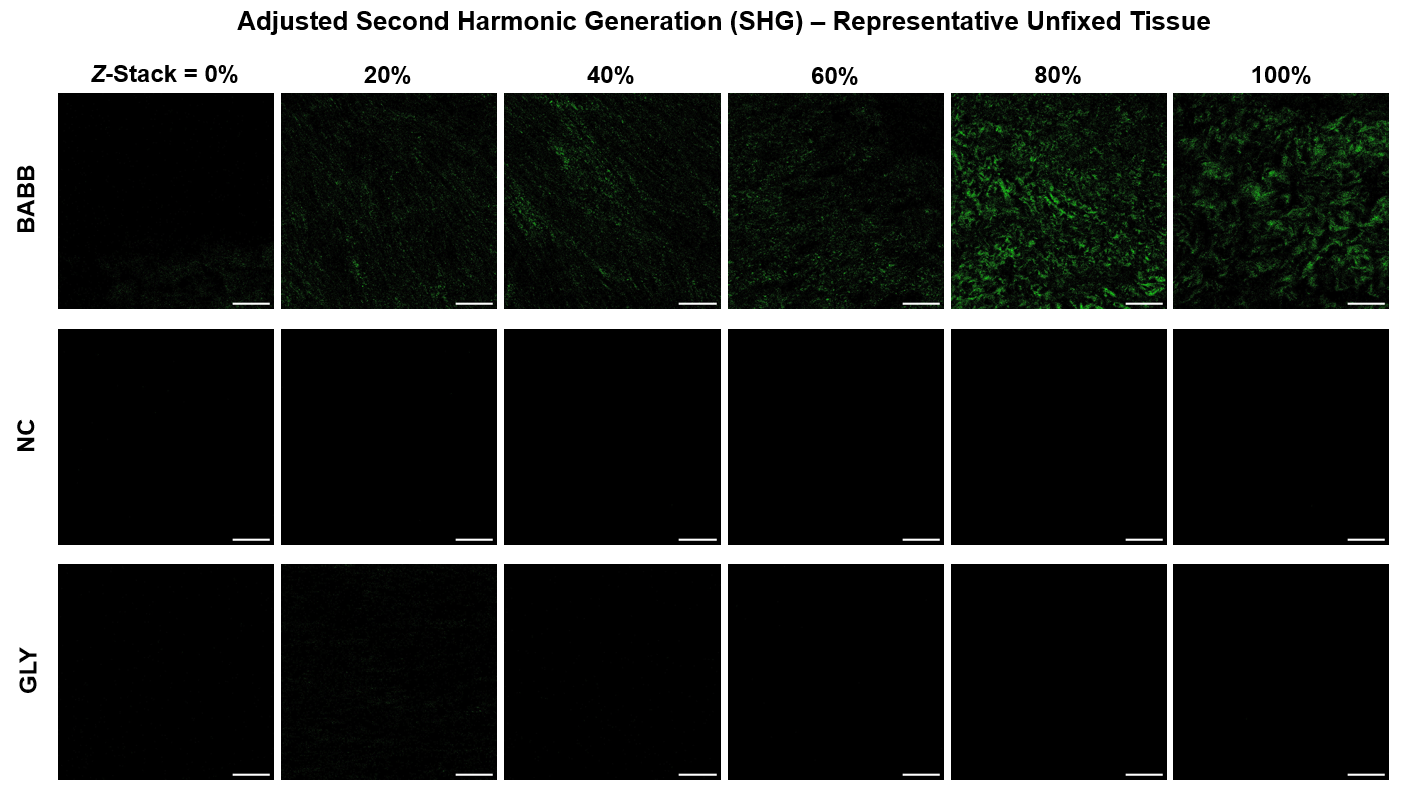


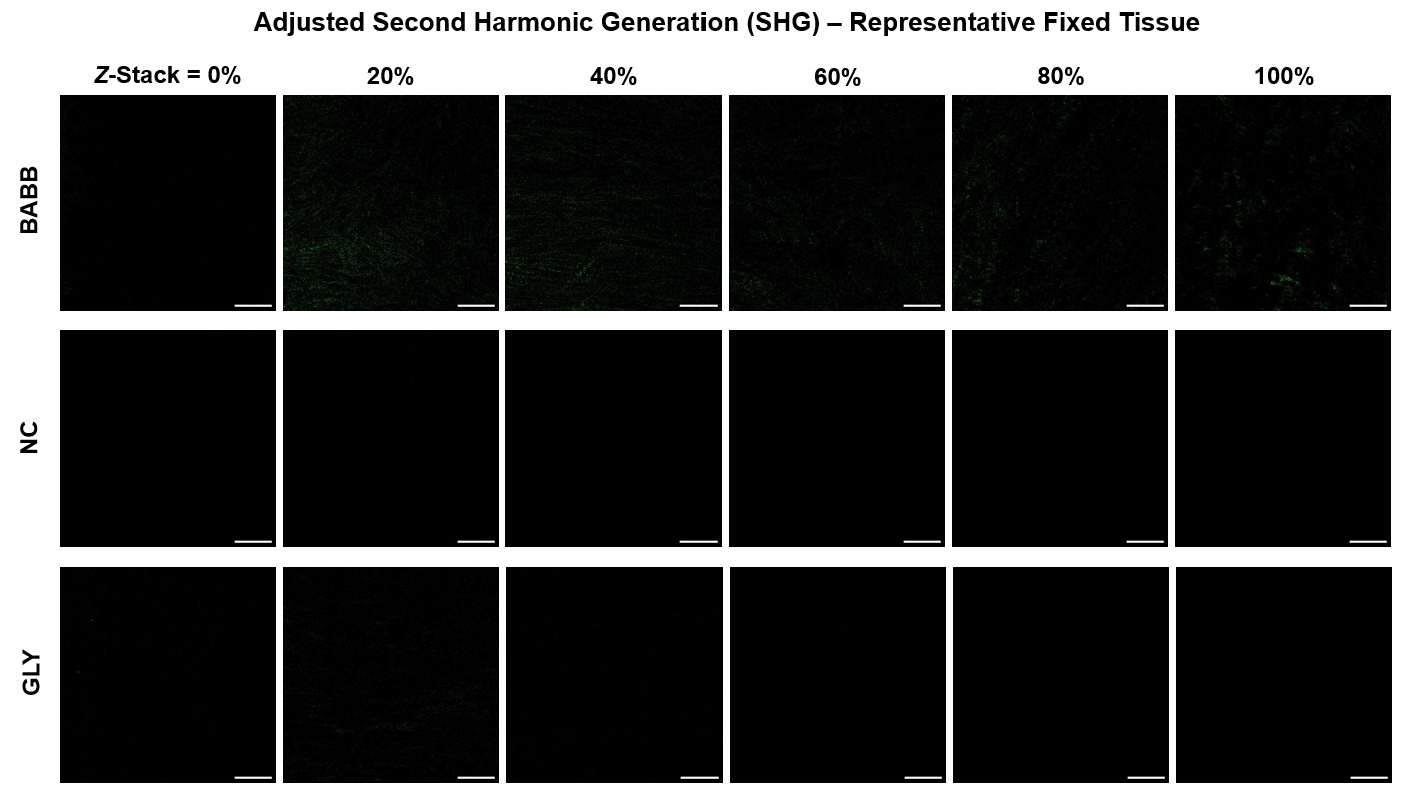


**Supplementary Figure 4.** Representative microscopic images of the second harmonic generation (SHG) signal vs. imaging depth for (*top row*) unfixed tissues and (*bottom row*) fixed tissues, cleared via BABB, glycerol (GLY), or uncleared (NC). Scale bars=50 µm. Brightness and contrast adjustments were performed on these images (min: 5, max: 15).

# Supplementary Tables

**Supplementary Table 1**: Summary of the specimen count and regional distribution of the three studies for autofluorescence (AF) and second harmonic generation (SHG) imaging.

| **Study** | **Group** | **Number of Specimens (**$N_{S}$**)** | **Number of Regions per Specimen (**$N_{R/S}$**)** | **Number of Regions in Total (**$N_{R}$**)** |
| --- | --- | --- | --- | --- |
| 1 | BABB | 4 | 3 | 12 |
| 1 | BABB-F | 3 | 3 | 9 |
| 1 | GLY | 4 | 3 | 12 |
| 1 | GLY-F | 3 | 3 | 9 |
| 1 | NC | 4 | 1 | 4 |
| 1 | NC-F | 4 | 1 | 4 |
| *Total Number of Experiments for Study 1 = 50* | | | | |
| 2 | F0 (same as BABB in Study 1) | 4 | 3 | 12 |
| 2 | F30 | 3 | 3 | 9 |
| 2 | F60 | 3 | 3 | 9 |
| 2 | F120 | 6 | 3 | 18 |
| 2 | F240 | 5 | 3 | 15 |
| *Total Number of Experiments for Study 2 = 63* | | | | |
| 3 | D0 | 3 | 3 | 9 |
| 3 | D1 | 3 | 3 | 9 |
| 3 | D3 | 3 | 3 | 9 |
| 3 | D7 | 3 | 3 | 9 |
| 3 | D14 | 3 | 3 | 9 |
| *Total Number of Experiments for Study 3 = 45* | | | | |

**Supplementary Table 2**: Comparisons of the area under the intensity curves between different pairs of groups in each study for autofluorescence (AF) and second harmonic generation (SHG). Only the statistically significant pairs (*p*<0.05) are reported here. See also **Fig. 2(b)** and **Fig. 3(b)** of the main text.

| **Study1** | | | | | | |  |
| --- | --- | --- | --- | --- | --- | --- | --- |
| **AF** | | | **SHG** | | | |  |
| Pairs | | *p*-value | Pairs | | | *p*-value |  |
| BABB-F vs. BABB | | 0.030 | BABB-F vs. NC | | | 0.030 |  |
| BABB-F vs. GLY | | 0.001 | BABB vs. GLY | | | 0.005 |  |
| BABB-F vs. NC-F | | 0.000 | BABB vs. NC-F | | | 0.003 |  |
| BABB-F vs. NC | | 0.000 | BABB vs. NC | | | 0.001 |  |
| BABB vs. GLY-F | | 0.001 |  | | |  |  |
| BABB vs. GLY | | 0.000 |  | | |  |  |
| BABB vs. NC-F | | 0.000 |  | | |  |  |
| BABB vs. NC | | 0.000 |  | | |  |  |
| GLY-F vs. GLY | | 0.031 |  | | |  |  |
| GLY-F vs. NC-F | | 0.000 |  | | |  |  |
| GLY-F vs. NC | | 0.000 |  | | |  |  |
| GLY vs. NC-F | | 0.007 |  | | |  |  |
| GLY vs. NC | | 0.000 |  | | |  |  |
| NC-F vs. NC | | 0.040 |  | | |  |  |
|  | |  |  | | |  |  |
| **Study2** | | | | | | | |
| **AF** | | | | **SHG** | | | |
| Pairs | *p*-value | | | Pairs | *p*-value | | |
| F120 vs. F0 | 0.035 | | | F120 vs. F0 | 0.048 | | |
| F120 vs. F30 | 0.015 | | |  |  | | |
| F120 vs. F60 | 0.044 | | |  |  | | |
| F240 vs. F120 | 0.007 | | |  |  | | |

**Supplementary Table 3**: Comparisons of the area under the intensity curves between BABB and BABB$-$F in each of the arterial layers (i.e., intima, media, adventitia) from Study 1 for autofluorescence (AF) and second harmonic generation (SHG) signals. Only the statistically significant pairs (*p*<0.05) are reported here.

| **Adventitia** | | | |
| --- | --- | --- | --- |
| **AF** | | **SHG** | |
| Pairs | *p*-value | Pairs | *p*-value |
| BABB vs. BABB-F | 0.022 | BABB vs. BABB-F | 0.004 |

**Supplementary Table 4**: Comparisons of tissue thicknesses within each study. Only the statistically significant pairs (*p*<0.05) are reported here.

| **Study 2** | | **Study 3** | |
| --- | --- | --- | --- |
| Pairs | *p*-value | Pairs | *p*-value |
| F0 vs. F240 | 0.034 | D0 vs. D7 | 0.003 |
| F120 vs. F240 | 0.034 | D1 vs. D7 | 0.001 |
|  |  | D3 vs. D7 | 0.036 |
|  |  | D1 vs. D14 | 0.031 |

**Supplementary Table 5**: Summary of individual sample characteristics with the median and interquartile range (IQR) for each characteristic within a group of Study 1 – autofluorescence (AF) signal.

| Group | ID | Tissue thickness (µm) | AUC | Z-stack with Max. Intensity (% of Tissue Thickness) | Max. Intensity | Imaging Depth  (% of tissue thickness) | Ratio of Min. to Max. Intensities  (%) | Avg. Intensity |
| --- | --- | --- | --- | --- | --- | --- | --- | --- |
| BABB | 1 | 320.0 | 0.118 | 64.0 | 0.160 | 100.0 | 24.0 | 0.118 |
|  | 2 | 324.0 | 0.134 | 55.8 | 0.181 | 100.0 | 34.3 | 0.133 |
|  | 3 | 366.0 | 0.138 | 34.2 | 0.218 | 100.0 | 26.7 | 0.137 |
|  | 4 | 342.0 | 0.140 | 4.7 | 0.256 | 100.0 | 21.9 | 0.140 |
|  | 5 | 340.0 | 0.078 | 7.6 | 0.148 | 100.0 | 6.0 | 0.078 |
|  | 6 | 324.0 | 0.104 | 50.3 | 0.196 | 100.0 | 14.3 | 0.105 |
|  | 7 | 376.0 | 0.121 | 64.6 | 0.209 | 100.0 | 24.8 | 0.121 |
|  | 8 | 462.0 | 0.114 | 57.8 | 0.161 | 100.0 | 32.5 | 0.114 |
|  | 9 | 420.0 | 0.124 | 53.1 | 0.227 | 100.0 | 25.7 | 0.124 |
|  | 10 | 364.0 | 0.123 | 2.7 | 0.201 | 100.0 | 15.7 | 0.122 |
|  | 11 | 274.0 | 0.123 | 68.1 | 0.171 | 100.0 | 19.0 | 0.123 |
|  | 12 | 328.0 | 0.130 | 45.5 | 0.202 | 100.0 | 26.6 | 0.129 |
| Median | | 341.0 | 0.123 | 51.7 | 0.199 | 100.0 | 24.4 | 0.123 |
| IQR | | 44.5 | 0.013 | 31.7 | 0.043 | 0.0 | 8.5 | 0.013 |
|  | | | | | | | | |
| GLY | 1 | 306.0 | 0.010 | 2.6 | 0.097 | 41.6 | 0.03 | 0.010 |
|  | 2 | 308.0 | 0.012 | 3.9 | 0.115 | 38.7 | 0.03 | 0.012 |
|  | 3 | 300.0 | 0.013 | 2.6 | 0.118 | 43.7 | 0.06 | 0.013 |
|  | 4 | 308.0 | 0.014 | 11.6 | 0.086 | 51.6 | 0.10 | 0.014 |
|  | 5 | 316.0 | 0.010 | 8.2 | 0.069 | 47.2 | 0.06 | 0.010 |
|  | 6 | 314.0 | 0.014 | 11.4 | 0.095 | 51.9 | 0.14 | 0.014 |
|  | 7 | 364.0 | 0.009 | 1.6 | 0.100 | 33.3 | 0.08 | 0.009 |
|  | 8 | 348.0 | 0.020 | 2.9 | 0.129 | 68.6 | 0.20 | 0.020 |
|  | 9 | 348.0 | 0.012 | 6.3 | 0.106 | 45.1 | 0.04 | 0.012 |
|  | 10 | 304.0 | 0.010 | 5.9 | 0.050 | 68.6 | 0.09 | 0.010 |
|  | 11 | 304.0 | 0.008 | 9.2 | 0.040 | 52.9 | 0.22 | 0.008 |
|  | 12 | 304.0 | 0.008 | 7.2 | 0.051 | 50.3 | 0.22 | 0.008 |
| Median | | 308.0 | 0.011 | 6.1 | 0.096 | 48.7 | 0.09 | 0.011 |
| IQR | | 20.0 | 0.004 | 5.6 | 0.044 | 9.0 | 0.10 | 0.004 |
|  | | | | | | | | |
| NC | 1 | 284.0 | 0.003 | 7.0 | 0.048 | 17.5 | 0.01 | 0.003 |
|  | 2 | 302.0 | 0.005 | 5.3 | 0.058 | 19.7 | 0.01 | 0.005 |
|  | 3 | 316.0 | 0.004 | 3.8 | 0.051 | 19.5 | 0.01 | 0.004 |
|  | 4 | 314.0 | 0.003 | 3.8 | 0.039 | 18.4 | 0.01 | 0.003 |
| Median | | 308.0 | 0.003 | 4.5 | 0.050 | 18.9 | 0.01 | 0.003 |
| IQR | | 17.0 | 0.001 | 1.9 | 0.007 | 1.4 | 0.00 | 0.001 |

| Group | ID | Tissue thickness (µm) | AUC | Z-stack with Max. Intensity (% of Tissue Thickness) | Max. Intensity | Imaging Depth  (% of tissue thickness) | Ratio of Min. to Max. Intensities (%) | Avg. Intensity |
| --- | --- | --- | --- | --- | --- | --- | --- | --- |
| BABB-F | 1 | 350.0 | 0.049 | 6.8 | 0.188 | 100.0 | 4.9 | 0.049 |
|  | 2 | 306.0 | 0.047 | 8.4 | 0.127 | 100.0 | 7.1 | 0.047 |
|  | 3 | 338.0 | 0.066 | 11.8 | 0.123 | 100.0 | 12.4 | 0.065 |
|  | 4 | 336.0 | 0.080 | 14.2 | 0.128 | 100.0 | 23.3 | 0.080 |
|  | 5 | 334.0 | 0.083 | 16.1 | 0.142 | 100.0 | 23.9 | 0.083 |
|  | 6 | 336.0 | 0.100 | 7.1 | 0.143 | 100.0 | 44.3 | 0.100 |
|  | 7 | 334.0 | 0.061 | 5.4 | 0.160 | 100.0 | 14.0 | 0.061 |
|  | 8 | 288.0 | 0.062 | 3.4 | 0.134 | 100.0 | 22.7 | 0.062 |
|  | 9 | 284.0 | 0.100 | 6.3 | 0.158 | 100.0 | 35.2 | 0.100 |
| Median | | 334.0 | 0.066 | 7.1 | 0.142 | 100.0 | 22.7 | 0.065 |
| IQR | | 30.0 | 0.022 | 5.5 | 0.029 | 0.0 | 11.5 | 0.022 |
|  | | | | | | | | |
| GLY-F | 1 | 348.0 | 0.070 | 2.9 | 0.251 | 61.7 | 0.08 | 0.070 |
|  | 2 | 352.0 | 0.066 | 7.9 | 0.208 | 63.3 | 0.03 | 0.065 |
|  | 3 | 350.0 | 0.063 | 6.8 | 0.232 | 60.2 | 0.02 | 0.063 |
|  | 4 | 352.0 | 0.032 | 11.9 | 0.099 | 72.9 | 0.04 | 0.032 |
|  | 5 | 352.0 | 0.029 | 7.3 | 0.092 | 72.9 | 0.15 | 0.029 |
|  | 6 | 316.0 | 0.040 | 11.3 | 0.105 | 77.4 | 0.11 | 0.040 |
|  | 7 | 322.0 | 0.034 | 8.6 | 0.101 | 82.1 | 0.58 | 0.033 |
|  | 8 | 322.0 | 0.032 | 7.4 | 0.151 | 71.0 | 0.04 | 0.032 |
|  | 9 | 320.0 | 0.037 | 6.8 | 0.118 | 81.4 | 0.41 | 0.037 |
| Median | | 348.0 | 0.037 | 7.4 | 0.118 | 72.9 | 0.08 | 0.037 |
| IQR | | 30.0 | 0.031 | 1.8 | 0.107 | 14.1 | 0.11 | 0.031 |
|  | | | | | | | | |
| NC-F | 1 | 284.0 | 0.004 | 3.5 | 0.065 | 19.6 | 0.01 | 0.004 |
|  | 2 | 302.0 | 0.007 | 3.9 | 0.097 | 17.1 | 0.00 | 0.007 |
|  | 3 | 302.0 | 0.008 | 0.7 | 0.091 | 23.7 | 0.01 | 0.009 |
|  | 4 | 286.0 | 0.004 | 3.5 | 0.073 | 16.0 | 0.01 | 0.004 |
| Median | | 294.0 | 0.005 | 3.5 | 0.082 | 18.3 | 0.01 | 0.005 |
| IQR | | 16.5 | 0.003 | 0.8 | 0.021 | 3.8 | 0.00 | 0.003 |

**Supplementary Table 6**: Summary of individual sample characteristics with the median and interquartile range (IQR) for each characteristic within a group of Study 1 – second harmonic generation (SHG) signal.

| Group | ID | Tissue thickness (µm) | AUC | Z-stack with Max. Intensity (% of Tissue Thickness) | Max. Intensity | Imaging Depth  (% of tissue thickness) | Ratio of Min. to Max. Intensities (%) | Avg. Intensity |
| --- | --- | --- | --- | --- | --- | --- | --- | --- |
| BABB | 1 | 320.0 | 0.007 | 71.4 | 0.012 | 100.0 | 11.0 | 0.007 |
|  | 2 | 324.0 | 0.008 | 84.0 | 0.013 | 100.0 | 13.3 | 0.008 |
|  | 3 | 366.0 | 0.016 | 60.3 | 0.040 | 100.0 | 6.1 | 0.016 |
|  | 4 | 342.0 | 0.004 | 95.9 | 0.009 | 100.0 | 4.2 | 0.004 |
|  | 5 | 340.0 | 0.004 | 88.9 | 0.008 | 100.0 | 1.2 | 0.004 |
|  | 6 | 324.0 | 0.003 | 69.3 | 0.006 | 100.0 | 2.6 | 0.003 |
|  | 7 | 376.0 | 0.004 | 100.0 | 0.013 | 100.0 | 8.2 | 0.004 |
|  | 8 | 462.0 | 0.005 | 100.0 | 0.015 | 100.0 | 13.0 | 0.005 |
|  | 9 | 420.0 | 0.009 | 95.3 | 0.021 | 100.0 | 8.9 | 0.009 |
|  | 10 | 364.0 | 0.012 | 69.4 | 0.023 | 100.0 | 8.3 | 0.012 |
|  | 11 | 274.0 | 0.005 | 87.7 | 0.007 | 100.0 | 15.8 | 0.005 |
|  | 12 | 328.0 | 0.009 | 70.3 | 0.021 | 100.0 | 8.0 | 0.009 |
| Median | | 341.0 | 0.006 | 85.9 | 0.013 | 100.0 | 8.2 | 0.006 |
| IQR | | 44.5 | 0.005 | 25.4 | 0.012 | 0.0 | 5.8 | 0.005 |
|  | | | | | | | | |
| GLY | 1 | 306.0 | 0.001 | 4.5 | 0.004 | 44.2 | 0.09 | 0.001 |
|  | 2 | 308.0 | 0.001 | 6.5 | 0.005 | 45.2 | 0.08 | 0.001 |
|  | 3 | 300.0 | 0.001 | 4.0 | 0.004 | 47.7 | 0.21 | 0.001 |
|  | 4 | 308.0 | 0.0004 | 19.4 | 0.001 | 76.8 | 1.31 | 0.0004 |
|  | 5 | 316.0 | 0.0004 | 15.7 | 0.001 | 57.2 | 0.56 | 0.0004 |
|  | 6 | 314.0 | 0.0004 | 10.1 | 0.001 | 77.8 | 1.21 | 0.0004 |
|  | 7 | 364.0 | 0.0003 | 3.3 | 0.001 | 95.6 | 4.42 | 0.0003 |
|  | 8 | 348.0 | 0.001 | 4.0 | 0.002 | 82.3 | 1.91 | 0.001 |
|  | 9 | 348.0 | 0.0004 | 8.0 | 0.002 | 45.7 | 0.39 | 0.0004 |
|  | 10 | 304.0 | 0.0004 | 13.7 | 0.002 | 66.0 | 0.52 | 0.0004 |
|  | 11 | 304.0 | 0.0003 | 16.3 | 0.001 | 59.5 | 0.85 | 0.0003 |
|  | 12 | 304.0 | 0.0003 | 9.8 | 0.001 | 56.9 | 0.81 | 0.0003 |
| Median | | 308.0 | 0.0004 | 8.9 | 0.002 | 58.4 | 0.69 | 0.0004 |
| IQR | | 20.0 | 0.0002 | 9.8 | 0.001 | 29.9 | 0.89 | 0.0002 |
|  | | | | | | | | |
| NC | 1 | 284.0 | 0.0001 | 5.6 | 0.0011 | 21.0 | 0.10 | 0.0001 |
|  | 2 | 302.0 | 0.0005 | 5.3 | 0.0047 | 24.3 | 0.03 | 0.0005 |
|  | 3 | 316.0 | 0.0004 | 3.1 | 0.0044 | 21.4 | 0.04 | 0.0004 |
|  | 4 | 314.0 | 0.0003 | 3.2 | 0.0036 | 20.3 | 0.03 | 0.0003 |
| Median | | 308.0 | 0.0004 | 4.2 | 0.0040 | 21.2 | 0.03 | 0.0004 |
| IQR | | 17.0 | 0.0002 | 2.2 | 0.0015 | 1.3 | 0.02 | 0.0002 |

|  | ID | Tissue thickness (µm) | AUC | Z-stack with Max. Intensity (% of Tissue Thickness) | Max. Intensity | Imaging Depth  (% of tissue thickness) | Ratio of Min. to Max. Intensities (%) | Avg. Intensity |
| --- | --- | --- | --- | --- | --- | --- | --- | --- |
| BABB-F | 1 | 350.0 | 0.003 | 50.0 | 0.004 | 100.0 | 9.8 | 0.003 |
|  | 2 | 306.0 | 0.002 | 52.6 | 0.003 | 100.0 | 19.8 | 0.002 |
|  | 3 | 338.0 | 0.003 | 50.6 | 0.005 | 100.0 | 20.6 | 0.003 |
|  | 4 | 336.0 | 0.004 | 32.0 | 0.006 | 100.0 | 35.2 | 0.004 |
|  | 5 | 334.0 | 0.004 | 25.0 | 0.006 | 100.0 | 13.5 | 0.004 |
|  | 6 | 336.0 | 0.004 | 35.5 | 0.006 | 100.0 | 43.0 | 0.004 |
|  | 7 | 334.0 | 0.003 | 56.5 | 0.005 | 100.0 | 20.8 | 0.003 |
|  | 8 | 288.0 | 0.004 | 82.8 | 0.005 | 100.0 | 51.4 | 0.004 |
|  | 9 | 284.0 | 0.005 | 66.4 | 0.006 | 100.0 | 38.1 | 0.005 |
| Median | | 334.0 | 0.004 | 50.6 | 0.005 | 100.0 | 20.8 | 0.004 |
| IQR | | 30.0 | 0.001 | 21.0 | 0.001 | 0.0 | 18.3 | 0.001 |
|  | | | | | | | | |
| GLY-F | 1 | 348.0 | 0.001 | 4.6 | 0.005 | 53.7 | 0.50 | 0.001 |
|  | 2 | 352.0 | 0.001 | 11.3 | 0.002 | 74.6 | 0.36 | 0.001 |
|  | 3 | 350.0 | 0.001 | 9.1 | 0.002 | 77.3 | 0.17 | 0.001 |
|  | 4 | 352.0 | 0.001 | 11.3 | 0.002 | 84.7 | 0.40 | 0.001 |
|  | 5 | 352.0 | 0.001 | 9.6 | 0.002 | 91.0 | 1.80 | 0.001 |
|  | 6 | 316.0 | 0.001 | 18.2 | 0.002 | 86.8 | 0.56 | 0.001 |
|  | 7 | 322.0 | 0.001 | 14.8 | 0.002 | 94.4 | 2.82 | 0.001 |
|  | 8 | 322.0 | 0.001 | 11.1 | 0.002 | 82.7 | 0.22 | 0.001 |
|  | 9 | 320.0 | 0.001 | 11.8 | 0.002 | 92.5 | 3.05 | 0.001 |
| Median | | 348.0 | 0.001 | 11.3 | 0.002 | 84.7 | 0.50 | 0.001 |
| IQR | | 30.0 | 0.000 | 2.2 | 0.0004 | 13.7 | 1.44 | 0.000 |
|  | | | | | | | | |
| NC-F | 1 | 284.0 | 0.004 | 3.5 | 0.066 | 19.6 | 0.01 | 0.004 |
|  | 2 | 302.0 | 0.0003 | 3.3 | 0.003 | 17.1 | 0.03 | 0.0003 |
|  | 3 | 302.0 | 0.0003 | 2.0 | 0.004 | 23.7 | 0.04 | 0.0003 |
|  | 4 | 286.0 | 0.0002 | 2.8 | 0.003 | 17.4 | 0.04 | 0.0002 |
| Median | | 294.0 | 0.0003 | 3.0 | 0.003 | 18.5 | 0.04 | 0.0003 |
| IQR | | 16.5 | 0.0009 | 0.8 | 0.016 | 3.3 | 0.02 | 0.0010 |

**Supplementary Table 7**: Summary of individual sample characteristics with the median and interquartile range (IQR) for each characteristic within a group of Study 2 – autofluorescence (AF) signal.

| Group | ID | Tissue thickness (µm) | AUC | Z-stack with Max. Intensity (% of Tissue Thickness) | Max. Intensity | Imaging Depth  (% of tissue thickness) | Ratio of Min. to Max. Intensities (%) | Avg. Intensity |
| --- | --- | --- | --- | --- | --- | --- | --- | --- |
| F0 | 1 | 320.0 | 0.118 | 64.0 | 0.160 | 100.0 | 24.0 | 0.118 |
|  | 2 | 324.0 | 0.134 | 55.8 | 0.181 | 100.0 | 34.3 | 0.133 |
|  | 3 | 366.0 | 0.138 | 34.2 | 0.218 | 100.0 | 26.7 | 0.137 |
|  | 4 | 342.0 | 0.140 | 4.7 | 0.256 | 100.0 | 21.9 | 0.140 |
|  | 5 | 340.0 | 0.078 | 7.6 | 0.148 | 100.0 | 6.04 | 0.078 |
|  | 6 | 324.0 | 0.104 | 50.3 | 0.196 | 100.0 | 14.3 | 0.105 |
|  | 7 | 376.0 | 0.121 | 64.6 | 0.209 | 100.0 | 24.8 | 0.121 |
|  | 8 | 462.0 | 0.114 | 57.8 | 0.161 | 100.0 | 32.5 | 0.114 |
|  | 9 | 420.0 | 0.124 | 53.1 | 0.227 | 100.0 | 25.7 | 0.124 |
|  | 10 | 364.0 | 0.123 | 2.7 | 0.201 | 100.0 | 15.7 | 0.122 |
|  | 11 | 274.0 | 0.123 | 68.1 | 0.171 | 100.0 | 19.0 | 0.123 |
|  | 12 | 328.0 | 0.130 | 45.5 | 0.202 | 100.0 | 26.6 | 0.129 |
| Median | | 341.0 | 0.123 | 51.7 | 0.199 | 100.0 | 24.4 | 0.123 |
| IQR | | 44.5 | 0.013 | 31.7 | 0.043 | 0.0 | 8.48 | 0.013 |
|  | | | | | | | | |
| F30 | 1 | 304.0 | 0.127 | 58.2 | 0.225 | 100.0 | 32.3 | 0.126 |
|  | 2 | 328.0 | 0.120 | 50.3 | 0.209 | 100.0 | 33.9 | 0.120 |
|  | 3 | 342.0 | 0.127 | 48.3 | 0.179 | 100.0 | 34.4 | 0.127 |
|  | 4 | 284.0 | 0.114 | 55.2 | 0.211 | 100.0 | 14.9 | 0.114 |
|  | 5 | 212.0 | 0.114 | 34.6 | 0.188 | 100.0 | 12.7 | 0.113 |
|  | 6 | 296.0 | 0.144 | 59.1 | 0.253 | 100.0 | 23.0 | 0.144 |
|  | 7 | 364.0 | 0.133 | 44.3 | 0.289 | 100.0 | 16.9 | 0.133 |
|  | 8 | 362.0 | 0.178 | 55.5 | 0.281 | 100.0 | 26.4 | 0.178 |
|  | 9 | 340.0 | 0.153 | 4.1 | 0.217 | 100.0 | 27.9 | 0.153 |
| Median | | 328.0 | 0.127 | 50.3 | 0.217 | 100.0 | 26.4 | 0.127 |
| IQR | | 46.0 | 0.024 | 11.2 | 0.045 | 0.0 | 15.4 | 0.024 |
|  | | | | | | | | |
| F60 | 1 | 346.0 | 0.085 | 34.5 | 0.149 | 100.0 | 20.8 | 0.085 |
|  | 2 | 334.0 | 0.073 | 45.8 | 0.124 | 100.0 | 28.1 | 0.073 |
|  | 3 | 276.0 | 0.079 | 52.5 | 0.139 | 100.0 | 20.5 | 0.079 |
|  | 4 | 384.0 | 0.151 | 57.0 | 0.245 | 100.0 | 9.55 | 0.151 |
|  | 5 | 388.0 | 0.172 | 57.4 | 0.285 | 100.0 | 6.69 | 0.171 |
|  | 6 | 364.0 | 0.169 | 4.4 | 0.267 | 100.0 | 29.5 | 0.169 |
|  | 7 | 356.0 | 0.108 | 46.4 | 0.235 | 100.0 | 21.8 | 0.108 |
|  | 8 | 328.0 | 0.123 | 57.6 | 0.247 | 100.0 | 15.7 | 0.123 |
|  | 9 | 396.0 | 0.138 | 62.8 | 0.187 | 100.0 | 39.1 | 0.138 |
| Median | | 356.0 | 0.123 | 52.5 | 0.235 | 100.0 | 20.8 | 0.123 |
| IQR | | 50.0 | 0.066 | 11.6 | 0.099 | 0.0 | 12.4 | 0.066 |

| Group | ID | Tissue thickness (µm) | AUC | Z-stack with Max. Intensity (% of Tissue Thickness) | Max. Intensity | Imaging Depth  (% of tissue thickness) | Ratio of Min. to Max. Intensities (%) | Avg. Intensity |
| --- | --- | --- | --- | --- | --- | --- | --- | --- |
| F120 | 1 | 332.0 | 0.077 | 44.3 | 0.146 | 100.0 | 35.7 | 0.077 |
|  | 2 | 302.0 | 0.085 | 41.4 | 0.153 | 100.0 | 40.7 | 0.085 |
|  | 3 | 336.0 | 0.077 | 40.2 | 0.128 | 100.0 | 41.4 | 0.077 |
|  | 4 | 414.0 | 0.046 | 50.5 | 0.109 | 100.0 | 20.1 | 0.046 |
|  | 5 | 432.0 | 0.044 | 55.8 | 0.107 | 100.0 | 13.0 | 0.044 |
|  | 6 | 374.0 | 0.042 | 71.3 | 0.092 | 100.0 | 22.7 | 0.042 |
|  | 7 | 386.0 | 0.023 | 62.9 | 0.050 | 100.0 | 27.2 | 0.023 |
|  | 8 | 400.0 | 0.021 | 72.1 | 0.051 | 100.0 | 23.1 | 0.021 |
|  | 9 | 384.0 | 0.031 | 70.5 | 0.059 | 100.0 | 31.4 | 0.031 |
|  | 10 | 350.0 | 0.049 | 6.8 | 0.188 | 100.0 | 4.94 | 0.049 |
|  | 11 | 306.0 | 0.047 | 8.4 | 0.127 | 100.0 | 7.08 | 0.047 |
|  | 12 | 338.0 | 0.066 | 11.8 | 0.123 | 100.0 | 12.4 | 0.065 |
|  | 13 | 336.0 | 0.080 | 14.2 | 0.128 | 100.0 | 23.3 | 0.080 |
|  | 14 | 334.0 | 0.083 | 16.1 | 0.142 | 100.0 | 23.9 | 0.083 |
|  | 15 | 336.0 | 0.100 | 7.1 | 0.143 | 100.0 | 44.3 | 0.100 |
|  | 16 | 334.0 | 0.061 | 5.4 | 0.160 | 100.0 | 14.0 | 0.061 |
|  | 17 | 288.0 | 0.062 | 3.4 | 0.134 | 100.0 | 22.7 | 0.062 |
|  | 18 | 284.0 | 0.100 | 6.3 | 0.158 | 100.0 | 35.2 | 0.100 |
| Median | | 336.0 | 0.062 | 28.2 | 0.128 | 100.0 | 23.2 | 0.062 |
| IQR | | 49.0 | 0.035 | 47.0 | 0.038 | 0.0 | 18.7 | 0.035 |
|  | | | | | | | | |
| F240 | 1 | 264.0 | 0.131 | 49.6 | 0.241 | 100.0 | 8.72 | 0.130 |
|  | 2 | 262.0 | 0.191 | 43.9 | 0.303 | 100.0 | 7.56 | 0.189 |
|  | 3 | 274.0 | 0.162 | 31.9 | 0.256 | 100.0 | 12.8 | 0.161 |
|  | 4 | 264.0 | 0.152 | 34.6 | 0.249 | 100.0 | 18.1 | 0.151 |
|  | 5 | 232.0 | 0.164 | 41.9 | 0.277 | 100.0 | 6.66 | 0.162 |
|  | 6 | 268.0 | 0.083 | 60.7 | 0.117 | 100.0 | 28.4 | 0.083 |
|  | 7 | 270.0 | 0.106 | 31.6 | 0.176 | 100.0 | 20.2 | 0.106 |
|  | 8 | 242.0 | 0.152 | 32.0 | 0.279 | 100.0 | 9.63 | 0.151 |
|  | 9 | 276.0 | 0.100 | 24.5 | 0.154 | 100.0 | 28.2 | 0.099 |
|  | 10 | 362.0 | 0.152 | 51.1 | 0.201 | 100.0 | 38.2 | 0.152 |
|  | 11 | 336.0 | 0.167 | 29.0 | 0.229 | 100.0 | 45.0 | 0.167 |
|  | 12 | 368.0 | 0.173 | 28.1 | 0.241 | 100.0 | 37.3 | 0.173 |
|  | 13 | 338.0 | 0.158 | 5.9 | 0.276 | 100.0 | 28.0 | 0.157 |
|  | 14 | 354.0 | 0.153 | 22.5 | 0.231 | 100.0 | 32.7 | 0.153 |
|  | 15 | 392.0 | 0.186 | 20.3 | 0.266 | 100.0 | 41.3 | 0.186 |
| Median | | 274.0 | 0.153 | 31.9 | 0.241 | 100.0 | 27.9 | 0.153 |
| IQR | | 82.0 | 0.024 | 16.6 | 0.055 | 0.0 | 23.8 | 0.024 |

**Supplementary Table 8**: Summary of individual sample characteristics with the median and interquartile range (IQR) for each characteristic within a group of Study 2 – second harmonic generation (SHG) signal.

| Group | ID | Tissue thickness (µm) | AUC | Z-stack with Max. Intensity (% of Tissue Thickness) | Max. Intensity | Imaging Depth  (% of tissue thickness) | Ratio of Min. to Max. Intensities (%) | Avg. Intensity |
| --- | --- | --- | --- | --- | --- | --- | --- | --- |
| F0 | 1 | 320.0 | 0.007 | 71.4 | 0.012 | 100.0 | 11.0 | 0.007 |
|  | 2 | 324.0 | 0.008 | 84.0 | 0.013 | 100.0 | 13.3 | 0.008 |
|  | 3 | 366.0 | 0.016 | 60.3 | 0.040 | 100.0 | 6.14 | 0.016 |
|  | 4 | 342.0 | 0.004 | 95.9 | 0.009 | 100.0 | 4.23 | 0.004 |
|  | 5 | 340.0 | 0.004 | 88.9 | 0.008 | 100.0 | 1.20 | 0.004 |
|  | 6 | 324.0 | 0.003 | 69.3 | 0.006 | 100.0 | 2.56 | 0.003 |
|  | 7 | 376.0 | 0.004 | 100.0 | 0.013 | 100.0 | 8.15 | 0.004 |
|  | 8 | 462.0 | 0.005 | 100.0 | 0.015 | 100.0 | 13.0 | 0.005 |
|  | 9 | 420.0 | 0.009 | 95.3 | 0.021 | 100.0 | 8.87 | 0.009 |
|  | 10 | 364.0 | 0.012 | 69.4 | 0.023 | 100.0 | 8.30 | 0.012 |
|  | 11 | 274.0 | 0.005 | 87.7 | 0.007 | 100.0 | 15.8 | 0.005 |
|  | 12 | 328.0 | 0.009 | 70.3 | 0.021 | 100.0 | 8.04 | 0.009 |
| Median | | 341.0 | 0.123 | 51.7 | 0.199 | 100.0 | 24.4 | 0.123 |
| IQR | | 44.5 | 0.013 | 31.7 | 0.043 | 0.0 | 8.48 | 0.013 |
|  | | | | | | | | |
| F30 | 1 | 304.0 | 0.007 | 89.5 | 0.014 | 100.0 | 7.54 | 0.007 |
|  | 2 | 328.0 | 0.008 | 97.0 | 0.014 | 100.0 | 10.5 | 0.008 |
|  | 3 | 342.0 | 0.010 | 78.5 | 0.016 | 100.0 | 10.0 | 0.010 |
|  | 4 | 284.0 | 0.004 | 40.6 | 0.009 | 100.0 | 3.24 | 0.004 |
|  | 5 | 212.0 | 0.004 | 15.0 | 0.008 | 100.0 | 2.18 | 0.004 |
|  | 6 | 296.0 | 0.006 | 49.7 | 0.013 | 100.0 | 6.72 | 0.006 |
|  | 7 | 364.0 | 0.011 | 80.9 | 0.023 | 100.0 | 2.23 | 0.011 |
|  | 8 | 362.0 | 0.008 | 100.0 | 0.019 | 100.0 | 6.33 | 0.008 |
|  | 9 | 340.0 | 0.010 | 90.6 | 0.022 | 100.0 | 3.64 | 0.010 |
| Median | | 328.0 | 0.008 | 80.9 | 0.014 | 100.0 | 6.33 | 0.008 |
| IQR | | 46.0 | 0.003 | 41.0 | 0.006 | 0.0 | 4.31 | 0.003 |
|  | | | | | | | | |
| F60 | 1 | 346.0 | 0.017 | 75.3 | 0.031 | 100.0 | 2.51 | 0.017 |
|  | 2 | 334.0 | 0.018 | 66.7 | 0.034 | 100.0 | 3.32 | 0.018 |
|  | 3 | 276.0 | 0.012 | 94.2 | 0.023 | 100.0 | 4.97 | 0.012 |
|  | 4 | 384.0 | 0.009 | 89.1 | 0.017 | 100.0 | 5.24 | 0.009 |
|  | 5 | 388.0 | 0.012 | 86.7 | 0.025 | 100.0 | 15.1 | 0.012 |
|  | 6 | 364.0 | 0.011 | 72.1 | 0.025 | 100.0 | 10.6 | 0.011 |
|  | 7 | 356.0 | 0.013 | 83.8 | 0.029 | 100.0 | 1.92 | 0.013 |
|  | 8 | 328.0 | 0.010 | 95.2 | 0.028 | 100.0 | 3.80 | 0.010 |
|  | 9 | 396.0 | 0.010 | 100.0 | 0.022 | 100.0 | 6.58 | 0.010 |
| Median | | 356.0 | 0.012 | 86.7 | 0.025 | 100.0 | 4.97 | 0.012 |
| IQR | | 50.0 | 0.003 | 19.0 | 0.006 | 0.0 | 3.26 | 0.003 |

| Group | ID | Tissue thickness (µm) | AUC | Z-stack with Max. Intensity (% of Tissue Thickness) | Max. Intensity | Imaging Depth  (% of tissue thickness) | Ratio of Min. to Max. Intensities (%) | Avg. Intensity |
| --- | --- | --- | --- | --- | --- | --- | --- | --- |
| F120 | 1 | 332.0 | 0.012 | 68.3 | 0.023 | 100.0 | 4.41 | 0.012 |
|  | 2 | 302.0 | 0.015 | 71.1 | 0.034 | 100.0 | 4.97 | 0.015 |
|  | 3 | 336.0 | 0.010 | 100.0 | 0.023 | 100.0 | 4.94 | 0.010 |
|  | 4 | 414.0 | 0.002 | 100.0 | 0.004 | 100.0 | 12.7 | 0.002 |
|  | 5 | 432.0 | 0.001 | 100.0 | 0.004 | 100.0 | 9.02 | 0.001 |
|  | 6 | 374.0 | 0.002 | 100.0 | 0.004 | 100.0 | 13.0 | 0.002 |
|  | 7 | 386.0 | 0.002 | 95.4 | 0.005 | 100.0 | 7.66 | 0.002 |
|  | 8 | 400.0 | 0.002 | 98.5 | 0.005 | 100.0 | 6.55 | 0.002 |
|  | 9 | 384.0 | 0.001 | 96.9 | 0.004 | 100.0 | 9.39 | 0.001 |
|  | 10 | 350.0 | 0.003 | 50.0 | 0.004 | 100.0 | 9.77 | 0.003 |
|  | 11 | 306.0 | 0.002 | 52.6 | 0.003 | 100.0 | 19.8 | 0.002 |
|  | 12 | 338.0 | 0.003 | 50.6 | 0.005 | 100.0 | 20.6 | 0.003 |
|  | 13 | 336.0 | 0.004 | 32.0 | 0.006 | 100.0 | 35.2 | 0.004 |
|  | 14 | 334.0 | 0.004 | 25.0 | 0.006 | 100.0 | 13.5 | 0.004 |
|  | 15 | 336.0 | 0.004 | 35.5 | 0.006 | 100.0 | 43.0 | 0.004 |
|  | 16 | 334.0 | 0.003 | 56.5 | 0.005 | 100.0 | 20.8 | 0.003 |
|  | 17 | 288.0 | 0.004 | 82.8 | 0.005 | 100.0 | 51.4 | 0.004 |
|  | 18 | 284.0 | 0.005 | 66.4 | 0.006 | 100.0 | 38.1 | 0.005 |
| Median | | 336.0 | 0.003 | 69.7 | 0.005 | 100.0 | 12.9 | 0.003 |
| IQR | | 49.0 | 0.002 | 47.0 | 0.001 | 0.0 | 12.7 | 0.002 |
|  | | | | | | | | |
| F240 | 1 | 264.0 | 0.009 | 94.0 | 0.020 | 100.0 | 2.81 | 0.009 |
|  | 2 | 262.0 | 0.009 | 96.2 | 0.024 | 100.0 | 2.00 | 0.009 |
|  | 3 | 274.0 | 0.011 | 100.0 | 0.032 | 100.0 | 1.51 | 0.011 |
|  | 4 | 264.0 | 0.011 | 97.0 | 0.025 | 100.0 | 3.05 | 0.011 |
|  | 5 | 232.0 | 0.007 | 90.6 | 0.019 | 100.0 | 1.55 | 0.007 |
|  | 6 | 268.0 | 0.008 | 100.0 | 0.019 | 100.0 | 6.23 | 0.008 |
|  | 7 | 270.0 | 0.011 | 97.1 | 0.028 | 100.0 | 4.47 | 0.011 |
|  | 8 | 242.0 | 0.009 | 89.3 | 0.018 | 100.0 | 2.79 | 0.009 |
|  | 9 | 276.0 | 0.013 | 100.0 | 0.025 | 100.0 | 4.72 | 0.013 |
|  | 10 | 362.0 | 0.007 | 75.8 | 0.012 | 100.0 | 13.2 | 0.007 |
|  | 11 | 336.0 | 0.007 | 100.0 | 0.016 | 100.0 | 7.63 | 0.007 |
|  | 12 | 368.0 | 0.004 | 100.0 | 0.007 | 100.0 | 22.0 | 0.004 |
|  | 13 | 338.0 | 0.005 | 97.6 | 0.009 | 100.0 | 15.3 | 0.005 |
|  | 14 | 354.0 | 0.005 | 98.3 | 0.007 | 100.0 | 26.0 | 0.005 |
|  | 15 | 392.0 | 0.006 | 100.0 | 0.017 | 100.0 | 10.2 | 0.006 |
| Median | | 274.0 | 0.008 | 97.6 | 0.019 | 100.0 | 4.7 | 0.007 |
| IQR | | 82.0 | 0.004 | 4.9 | 0.011 | 0.0 | 8.8 | 0.004 |

**Supplementary Table 9**: Summary of individual sample characteristics with the median and interquartile range (IQR) for each characteristic within a group of Study 3 – autofluorescence (AF) signal.

| Group | ID | Tissue thickness (µm) | AUC | Z-stack with Max. Intensity (% of Tissue Thickness) | Max. Intensity | Imaging Depth  (% of tissue thickness) | Ratio of Min. to Max. Intensities (%) | Avg. Intensity |
| --- | --- | --- | --- | --- | --- | --- | --- | --- |
| D0 | 1 | 358.0 | 0.056 | 73.3 | 0.086 | 100.0 | 15.0 | 0.056 |
|  | 2 | 312.0 | 0.063 | 58.0 | 0.101 | 100.0 | 18.1 | 0.063 |
|  | 3 | 268.0 | 0.065 | 68.9 | 0.114 | 100.0 | 16.1 | 0.064 |
|  | 4 | 272.0 | 0.055 | 75.9 | 0.081 | 100.0 | 15.1 | 0.055 |
|  | 5 | 210.0 | 0.071 | 65.1 | 0.111 | 100.0 | 21.7 | 0.070 |
|  | 6 | 232.0 | 0.067 | 62.4 | 0.106 | 100.0 | 27.5 | 0.067 |
|  | 7 | 350.0 | 0.049 | 8.5 | 0.077 | 100.0 | 35.0 | 0.048 |
|  | 8 | 336.0 | 0.052 | 3.6 | 0.086 | 100.0 | 28.9 | 0.052 |
|  | 9 | 350.0 | 0.055 | 13.6 | 0.072 | 100.0 | 52.8 | 0.055 |
| Median | | 312.0 | 0.056 | 62.4 | 0.086 | 100.0 | 21.7 | 0.055 |
| IQR | | 82.0 | 0.010 | 55.3 | 0.025 | 0.0 | 12.7 | 0.010 |
|  | | | | | | | | |
| D1 | 1 | 296.0 | 0.071 | 76.5 | 0.129 | 100.0 | 11.5 | 0.071 |
|  | 2 | 228.0 | 0.074 | 65.2 | 0.140 | 100.0 | 17.9 | 0.073 |
|  | 3 | 256.0 | 0.072 | 75.2 | 0.124 | 100.0 | 13.8 | 0.072 |
|  | 4 | 300.0 | 0.055 | 64.9 | 0.083 | 100.0 | 25.8 | 0.055 |
|  | 5 | 348.0 | 0.048 | 65.7 | 0.088 | 100.0 | 24.9 | 0.048 |
|  | 6 | 244.0 | 0.051 | 63.4 | 0.091 | 1000. | 17.9 | 0.051 |
|  | 7 | 334.0 | 0.044 | 66.1 | 0.066 | 100.0 | 34.7 | 0.044 |
|  | 8 | 336.0 | 0.043 | 9.5 | 0.067 | 100.0 | 26.5 | 0.043 |
|  | 9 | 394.0 | 0.045 | 2.0 | 0.073 | 100.0 | 41.6 | 0.045 |
| Median | | 300.0 | 0.051 | 65.2 | 0.088 | 100.0 | 24.9 | 0.051 |
| IQR | | 80.0 | 0.026 | 2.7 | 0.051 | 0.0 | 8.64 | 0.026 |
|  | | | | | | | | |
| D3 | 1 | 332.0 | 0.070 | 62.9 | 0.115 | 100.0 | 16.4 | 0.070 |
|  | 2 | 310.0 | 0.062 | 10.3 | 0.093 | 100.0 | 17.8 | 0.062 |
|  | 3 | 284.0 | 0.071 | 58.0 | 0.109 | 100.0 | 21.3 | 0.071 |
|  | 4 | 254.0 | 0.070 | 68.0 | 0.131 | 100.0 | 9.89 | 0.070 |
|  | 5 | 248.0 | 0.060 | 68.0 | 0.099 | 100.0 | 17.2 | 0.060 |
|  | 6 | 284.0 | 0.059 | 70.6 | 0.082 | 100.0 | 15.0 | 0.058 |
|  | 7 | 272.0 | 0.061 | 75.9 | 0.081 | 100.0 | 32.0 | 0.060 |
|  | 8 | 266.0 | 0.058 | 49.3 | 0.089 | 100.0 | 15.9 | 0.057 |
|  | 9 | 264.0 | 0.056 | 64.7 | 0.095 | 100.0 | 23.5 | 0.056 |
| Median | | 272.0 | 0.061 | 64.7 | 0.095 | 100.0 | 17.2 | 0.060 |
| IQR | | 20.0 | 0.012 | 10.0 | 0.020 | 0.0 | 5.40 | 0.012 |

| Group | ID | Tissue thickness (µm) | AUC | Z-stack with Max. Intensity (% of Tissue Thickness) | Max. Intensity | Imaging Depth  (% of tissue thickness) | Ratio of Min. to Max. Intensities (%) | Avg. Intensity |
| --- | --- | --- | --- | --- | --- | --- | --- | --- |
| D7 | 1 | 184.0 | 0.074 | 77.4 | 0.109 | 100.0 | 19.3 | 0.074 |
|  | 2 | 238.0 | 0.073 | 58.3 | 0.111 | 100.0 | 29.5 | 0.072 |
|  | 3 | 214.0 | 0.062 | 69.4 | 0.089 | 100.0 | 41.2 | 0.062 |
|  | 4 | 208.0 | 0.074 | 67.6 | 0.117 | 100.0 | 30.3 | 0.074 |
|  | 5 | 270.0 | 0.054 | 73.5 | 0.084 | 100.0 | 22.8 | 0.054 |
|  | 6 | 242.0 | 0.046 | 73.8 | 0.066 | 100.0 | 27.6 | 0.046 |
|  | 7 | 204.0 | 0.038 | 64.1 | 0.061 | 100.0 | 21.2 | 0.038 |
|  | 8 | 226.0 | 0.039 | 52.6 | 0.057 | 100.0 | 41.3 | 0.039 |
|  | 9 | 190.0 | 0.035 | 58.3 | 0.066 | 1000. | 19.8 | 0.035 |
| Median | | 214.0 | 0.054 | 67.6 | 0.084 | 100.0 | 27.6 | 0.054 |
| IQR | | 34.0 | 0.034 | 15.2 | 0.044 | 0.0 | 9.14 | 0.034 |
|  | | | | | | | | |
| D14 | 1 | 278.0 | 0.063 | 5.7 | 0.120 | 100.0 | 17.7 | 0.063 |
|  | 2 | 258.0 | 0.057 | 10.8 | 0.082 | 100.0 | 20.5 | 0.057 |
|  | 3 | 248.0 | 0.064 | 85.6 | 0.088 | 100.0 | 23.7 | 0.063 |
|  | 4 | 204.0 | 0.072 | 67.0 | 0.143 | 100.0 | 14.1 | 0.072 |
|  | 5 | 202.0 | 0.073 | 55.9 | 0.125 | 100.0 | 31.8 | 0.073 |
|  | 6 | 208.0 | 0.066 | 73.3 | 0.102 | 100.0 | 28.4 | 0.066 |
|  | 7 | 222.0 | 0.067 | 69.6 | 0.087 | 100.0 | 28.4 | 0.066 |
|  | 8 | 252.0 | 0.059 | 57.5 | 0.086 | 100.0 | 23.2 | 0.058 |
|  | 9 | 316.0 | 0.059 | 3.8 | 0.101 | 100.0 | 44.8 | 0.059 |
| Median | | 248.0 | 0.064 | 57.5 | 0.101 | 100.0 | 23.7 | 0.063 |
| IQR | | 50.0 | 0.007 | 58.9 | 0.033 | 0.0 | 7.84 | 0.007 |

**Supplementary Table 10**: Summary of individual sample characteristics with the median and interquartile range (IQR) for each characteristic within a group of Study 3 – second harmonic generation (SHG) signal.

| Group | ID | Tissue thickness (µm) | AUC | Z-stack with Max. Intensity (% of Tissue Thickness) | Max. Intensity | Imaging Depth  (% of tissue thickness) | Ratio of Min. to Max. Intensities (%) | Avg. Intensity |
| --- | --- | --- | --- | --- | --- | --- | --- | --- |
| D0 | 1 | 358.0 | 0.002 | 25.6 | 0.003 | 100.0 | 9.59 | 0.002 |
|  | 2 | 312.0 | 0.004 | 82.8 | 0.005 | 100.0 | 10.7 | 0.004 |
|  | 3 | 268.0 | 0.004 | 93.3 | 0.006 | 100.0 | 7.85 | 0.004 |
|  | 4 | 272.0 | 0.002 | 86.9 | 0.004 | 100.0 | 6.84 | 0.002 |
|  | 5 | 210.0 | 0.002 | 20.8 | 0.003 | 100.0 | 26.0 | 0.002 |
|  | 6 | 232.0 | 0.004 | 77.8 | 0.008 | 100.0 | 8.96 | 0.004 |
|  | 7 | 350.0 | 0.002 | 11.4 | 0.004 | 100.0 | 19.4 | 0.002 |
|  | 8 | 336.0 | 0.002 | 7.1 | 0.005 | 100.0 | 17.8 | 0.002 |
|  | 9 | 350.0 | 0.002 | 15.3 | 0.004 | 100.0 | 18.5 | 0.002 |
| Median | | 312.0 | 0.002 | 25.6 | 0.004 | 100.0 | 10.7 | 0.002 |
| IQR | | 82.0 | 0.002 | 67.5 | 0.001 | 0.0 | 9.55 | 0.002 |
|  | | | | | | | | |
| D1 | 1 | 296.0 | 0.004 | 92.6 | 0.012 | 100.0 | 2.67 | 0.004 |
|  | 2 | 228.0 | 0.004 | 93.9 | 0.009 | 100.0 | 8.05 | 0.004 |
|  | 3 | 256.0 | 0.003 | 99.2 | 0.011 | 100.0 | 3.18 | 0.003 |
|  | 4 | 300.0 | 0.004 | 87.4 | 0.007 | 100.0 | 22.3 | 0.004 |
|  | 5 | 348.0 | 0.004 | 88.6 | 0.009 | 100.0 | 9.62 | 0.004 |
|  | 6 | 244.0 | 0.006 | 96.7 | 0.016 | 100.0 | 3.42 | 0.006 |
|  | 7 | 334.0 | 0.003 | 100.0 | 0.008 | 100.0 | 11.5 | 0.003 |
|  | 8 | 336.0 | 0.003 | 85.2 | 0.006 | 100.0 | 11.6 | 0.003 |
|  | 9 | 394.0 | 0.002 | 5.1 | 0.004 | 100.0 | 10.0 | 0.002 |
| Median | | 300.0 | 0.004 | 92.6 | 0.009 | 100.0 | 9.62 | 0.004 |
| IQR | | 80.0 | 0.001 | 9.3 | 0.004 | 0 | 8.05 | 0.001 |
|  | | | | | | | | |
| D3 | 1 | 332.0 | 0.002 | 12.0 | 0.005 | 100.0 | 14.3 | 0.002 |
|  | 2 | 310.0 | 0.003 | 14.7 | 0.006 | 100.0 | 9.97 | 0.003 |
|  | 3 | 284.0 | 0.004 | 10.5 | 0.006 | 100.0 | 36.2 | 0.004 |
|  | 4 | 254.0 | 0.004 | 98.4 | 0.008 | 100.0 | 6.69 | 0.004 |
|  | 5 | 248.0 | 0.003 | 92.8 | 0.007 | 100.0 | 7.21 | 0.003 |
|  | 6 | 284.0 | 0.003 | 21.0 | 0.004 | 100.0 | 13.0 | 0.003 |
|  | 7 | 272.0 | 0.004 | 91.2 | 0.008 | 100.0 | 22.9 | 0.004 |
|  | 8 | 266.0 | 0.004 | 88.8 | 0.007 | 100.0 | 9.27 | 0.004 |
|  | 9 | 264.0 | 0.004 | 85.0 | 0.007 | 100.0 | 10.5 | 0.004 |
| Median | | 272.0 | 0.004 | 85.0 | 0.007 | 100.0 | 10.5 | 0.004 |
| IQR | | 20.0 | 0.001 | 76.5 | 0.002 | 0.0 | 5.06 | 0.002 |

| Group | ID | Tissue thickness (µm) | AUC | Z-stack with Max. Intensity (% of Tissue Thickness) | Max. Intensity | Imaging Depth  (% of tissue thickness) | Ratio of Min. to Max. Intensities (%) | Avg. Intensity |
| --- | --- | --- | --- | --- | --- | --- | --- | --- |
| D7 | 1 | 184.0 | 0.004 | 89.2 | 0.006 | 100.0 | 11.2 | 0.004 |
|  | 2 | 238.0 | 0.003 | 89.2 | 0.005 | 100.0 | 21.5 | 0.003 |
|  | 3 | 214.0 | 0.003 | 90.7 | 0.005 | 100.0 | 29.7 | 0.003 |
|  | 4 | 208.0 | 0.004 | 85.7 | 0.006 | 100.0 | 26.3 | 0.004 |
|  | 5 | 270.0 | 0.002 | 99.3 | 0.003 | 100.0 | 24.7 | 0.002 |
|  | 6 | 242.0 | 0.003 | 88.5 | 0.005 | 100.0 | 16.0 | 0.003 |
|  | 7 | 204.0 | 0.004 | 78.6 | 0.005 | 100.0 | 11.1 | 0.004 |
|  | 8 | 226.0 | 0.003 | 21.1 | 0.003 | 100.0 | 31.2 | 0.003 |
|  | 9 | 190.0 | 0.003 | 74.0 | 0.004 | 100.0 | 17.9 | 0.003 |
| Median | | 214.0 | 0.003 | 88.5 | 0.005 | 100.0 | 21.5 | 0.003 |
| IQR | | 34.0 | 0.001 | 10.6 | 0.001 | 0.0 | 10.3 | 0.001 |
|  | | | | | | | | |
| D14 | 1 | 278.0 | 0.003 | 70.7 | 0.005 | 100.0 | 17.7 | 0.003 |
|  | 2 | 258.0 | 0.003 | 100.0 | 0.006 | 100.0 | 6.61 | 0.003 |
|  | 3 | 248.0 | 0.003 | 99.2 | 0.007 | 100.0 | 5.42 | 0.003 |
|  | 4 | 204.0 | 0.005 | 96.1 | 0.010 | 100.0 | 6.94 | 0.005 |
|  | 5 | 202.0 | 0.007 | 96.1 | 0.013 | 100.0 | 17.9 | 0.007 |
|  | 6 | 208.0 | 0.004 | 100.0 | 0.011 | 100.0 | 8.68 | 0.004 |
|  | 7 | 222.0 | 0.002 | 10.7 | 0.003 | 100.0 | 32.6 | 0.002 |
|  | 8 | 252.0 | 0.002 | 11.0 | 0.003 | 100.0 | 21.3 | 0.002 |
|  | 9 | 316.0 | 0.003 | 99.4 | 0.005 | 100.0 | 26.5 | 0.003 |
| Median | | 248.0 | 0.003 | 96.1 | 0.006 | 100.0 | 17.7 | 0.003 |
| IQR | | 50.0 | 0.002 | 28.7 | 0.010 | 0.0 | 14.3 | 0.002 |

**Supplementary Table 11**: Summary of individual sample characteristics with the median and interquartile range (IQR) for each characteristic within a group of Study 4 – autofluorescence (AF) signal.

| Group | ID | Tissue thickness (µm) | AUC | Z-stack with Max. Intensity (% of Tissue Thickness) | Max. Intensity | Imaging Depth  (% of tissue thickness) | Ratio of Min. to Max. Intensities (%) | Avg. Intensity |
| --- | --- | --- | --- | --- | --- | --- | --- | --- |
| S1 | 1 | 290.0 | 0.013 | 6.8 | 0.061 | 100.0 | 7.08 | 0.013 |
| S2 | 1 | 272.0 | 0.007 | 10.2 | 0.048 | 100.0 | 0.37 | 0.007 |
| S3 | 1 | 352.0 | 0.040 | 50.8 | 0.112 | 100.0 | 14.3 | 0.040 |
| S4 | 1 | 414.0 | 0.030 | 65.4 | 0.070 | 28.4 | 21.0 | 0.030 |

**Supplementary Table 12**: Summary of individual sample characteristics with the median and interquartile range (IQR) for each characteristic within a group of Study 4 – second harmonic generation (SHG) signal.

| Group | ID | Tissue thickness (µm) | AUC | Z-stack with Max. Intensity (% of Tissue Thickness) | Max. Intensity | Imaging Depth  (% of tissue thickness) | Ratio of Min. to Max. Intensities (%) | Avg. Intensity |
| --- | --- | --- | --- | --- | --- | --- | --- | --- |
| S1 | 1 | 290.0 | 0.001 | 13.0 | 0.002 | 100.0 | 0.159 | 0.001 |
| S2 | 1 | 272.0 | 0.001 | 10.2 | 0.003 | 100.0 | 0.005 | 0.001 |
| S3 | 1 | 352.0 | 0.007 | 100.0 | 0.019 | 100.0 | 0.127 | 0.007 |
| S4 | 1 | 414.0 | 0.004 | 76.9 | 0.008 | 37.0 | 0.193 | 0.004 |
