# Supplementary material for: Evaluation of the effects of clearing agents, fixation, and process durations on cardiovascular tissue imaging with second harmonic generation and multi-photon modalities
Source: Front Bioeng Biotechnol. 2025 Jul 25;13:1606425. doi: 10.3389/fbioe.2025.1606425 (PMC12331724; doi:10.3389/fbioe.2025.1606425)
Supplement: Supplementary file 2 [file Supplementaryfile2.docx]

Supplementary Material (Supplementary Appendices)

This Supplementary Material section provides supplementary appendices for the manuscript “*Evaluation of the effects of clearing agents, fixation, and process durations on cardiovascular tissue imaging with second harmonic generation and multi-photon modalities*,” authored by Maedeh Makki, Zachary A. Molander, Sergio A. Pineda-Castillo, Devin W. Laurence, Shubhra Singhal, Yasmin Eltwafsha, Gerhard A. Holzapfel, Tingting Gu, Chung-Hao Lee.

# Supplementary Appendix A: Reproducing Kernel Interpolation of Tissue Intensity Data – Overview on Key Concepts and Equations

An in-house reproducing kernel (RK)-based interpolation algorithm was implemented in MATLAB to interpolate the intensity data and thus resolve inconsistencies in tissue thickness (**Section 2.6** of the main manuscript). The following briefly reviews the RK shape function and its use for function approximation and to generate intensity values for consistent tissue depths in different imaged tissue samples.

For a given intensity $y_{J}$ at tissue depth $x_{J}$ measured from MPM imaging, an intensity function *y*(*x*) can be constructed as follows:

| $y\left( x \right)=\sum_{I=1}^{NP} \Phi_{I}\left( x \right)d_{I},$ | $(A.1)$ |
| --- | --- |

where $NP$ is the number of RK points used to construct the function approximation, and $\Phi_{I}\left( x \right)$ is the *I*^th^ RK point with its nodal value $d_{I}$. According to Chen et al. (1996), the RK shape function has the form

| $\Phi_{I}\left( x \right)=\phi_{a}\left( x - x_{I} \right)\mathbf{H}^{T}\left( 0 \right)\mathbf{M}^{-1}\left( x \right)\mathbf{H}\left( x - x_{I} \right),$ | $(A.2)$ |
| --- | --- |

where $\mathbf{H}\left( x \right)$ is the monomial basis function up to order *p*, i.e.,

| $\mathbf{H}(x) = \left[ \begin{matrix} 1 \\ x \\ \vdots\\ x^{p} \end{matrix} \right]$, | $(A.3)$ |
| --- | --- |

$\phi_{a}\left( x - x_{I} \right)$ is the kernel function with a local support size of $a$ to control the locality and smoothness of the function:

| $\phi_{a}\left( x - x_{I} \right)=\phi\left( \frac{\left\vert x - x_{I} \right\vert}{a} \right) \mathrm{and}$  $\phi(z) =\left\{ \begin{matrix} 0, & z\geq1 \\ \frac{4}{3} - 4z + 4z^{2} - \frac{4}{3}z^{3}, & 0.5\leq z<1, \\ \frac{2}{3} - 4{z_{r}}^{2} + 4{z_{r}}^{3}, & 0\leq z<0.5 \end{matrix} \right.$ | $(A.4)$ |
| --- | --- |

and $\mathbf{M}\left( x \right)$ is the moment matrix determined by enforcing the reproducing conditions of polynomials up to order *p*$:$​

| $\mathbf{M}\left( x \right)= \sum_{I=1}^{NP} \phi_{a}\left( x - x_{I} \right)\mathbf{H}\left( x - x_{I} \right)\mathbf{H}^{\boldsymbol{T}}\left( x - x_{I} \right).$ | $(A.5)$ |
| --- | --- |

In this study, we used 51 uniformly spaced RK points between normalized tissue depth of $0-1$, a linear basis (i.e. $p=1$), and a support size $a=3.91\times0.02$. The unknown coefficients $d_{I}$ were determined using the least squares method on the given data points $\left( x_{J},y_{J} \right), J=1\sim n$. After solving the RK coefficients $d_{I}$, we next generated 201 uniformly spaced points between a normalized tissue depth of 0-1 and calculated their corresponding intensity values using RK approximation in Eq. (A.1). This provided consistent grid points that can be used to determine the study-average intensity responses, as shown in **Fig. 2(a)**, **Fig. 4(a)**, and **Fig. 5(a)**.

# Supplementary Appendix B: Normalized Intensity Across Normalized Depth for Pilot Comparative Study

To assess the effect of sequential tissue clearing in the four graded clearing solutions (combinations of BABB and ethanol), we performed imaging and intensity data analysis after each 30-minute clearing step. This exploratory study was performed on one region from four different specimens and analyzed based on the AF and SHG signals at four consecutive clearing steps over a total duration of 120 minutes. Due to the limited number of experiments, no statistical analysis was performed for this pilot comparative study. Nevertheless, visualizations of the normalized intensity across normalized depth for AF and SHG signals showed an increased intensity after clearing in 100% BABB during Steps 3 and 4, particularly beyond half the specimen thickness ($z$/$z_{\max}>0.5$). Further, extending the clearing process in 100% BABB for an additional 30 minutes after Step 3, resulted in higher AF intensity near full depth compared to Step 3 alone, while simultaneously reducing the SHG signal (**Fig. A1**).

**Remark:** The individual sample characteristics (e.g., tissue thickness (µm), AUC, maximum intensity, etc.) for each characteristic within each group of Study 4, are presented in the **Supplementary Tables 11 and 12, *Supplementary Material*** for both the AF and SHG signals, respectively.

This Supplementary Material section provides supplementary tables and figures for the manuscript “*Evaluation of the effects of clearing agents, fixation, and process durations on cardiovascular tissue imaging with second harmonic generation and multi-photon modalities*,” authored by Maedeh Makki, Zachary A. Molander, Sergio A. Pineda-Castillo, Devin W. Laurence, Shubhra Singhal, Yasmin Eltwafsha, Gerhard A. Holzapfel, Tingting Gu, Chung-Hao Lee.

In this section, the comparison of the area under the normalized intensity curves of the arterial layers (intima, media, adventitia) for BABB and BABB-F from Study 1 and the comparison of the sample thicknesses among the test groups in each study are shown in **Supplementary Figure 1** and **Supplementary Figure 2**, respectively. **Supplementary Figure 3 & 4** present the representative images throughout the tissue to visually contrast the clearing techniques in Study 1.

In addition, **Supplementary Table 1** contains the summary of specimen and region distribution across studies. **Supplementary Table 2** shows the *p*-values for comparisons between groups in each of the three studies (see Table 1 in the main text). **Supplementary Table 3** lists the *p*-values for comparisons between BABB, BABB-F in the arterial layers of Study 1. **Supplementary Table 4** summarizes the *p*-values for the comparison of sample thicknesses among different groups in each study. **Supplementary Tables 5-12** show individual sample characteristics.

# Supplementary Appendix Figure


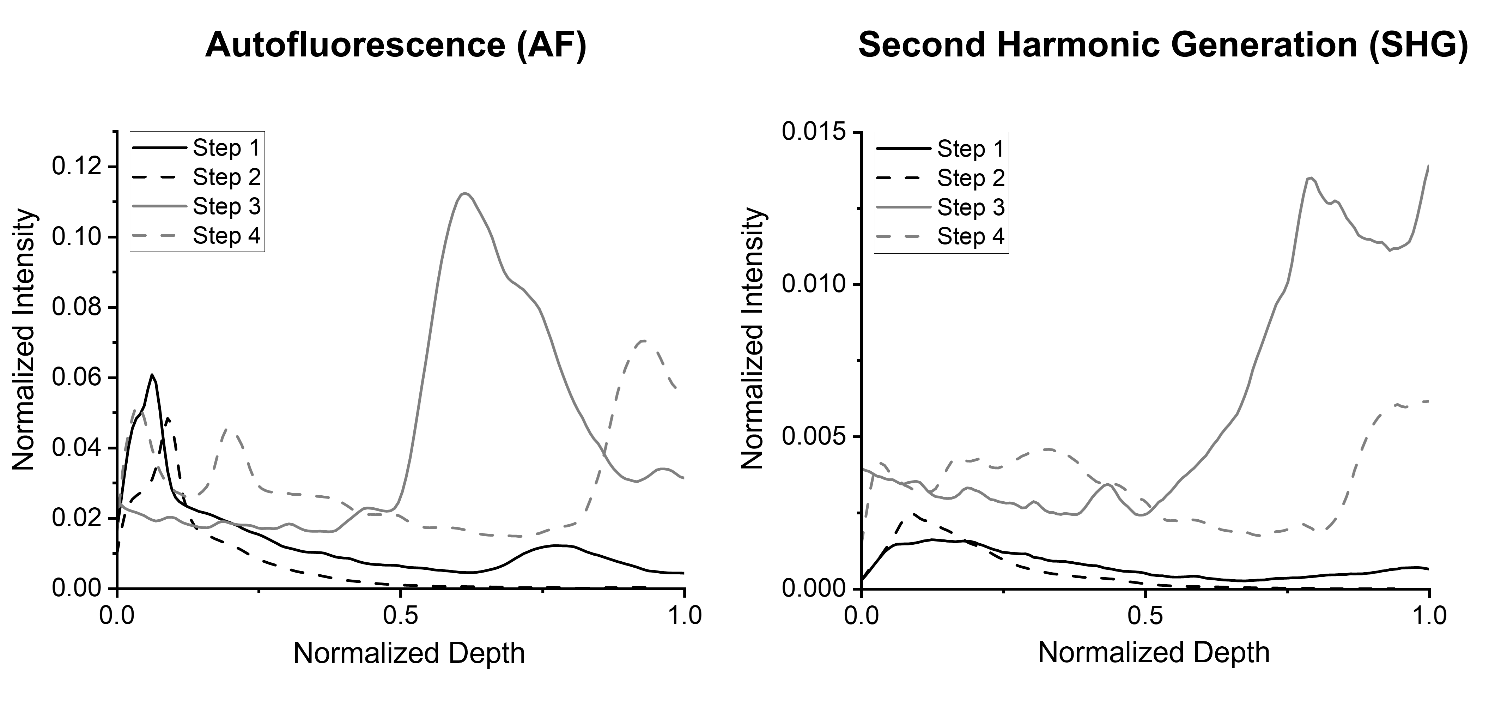
 **Supplementary Figure A1.** Investigation of the effects of each clearing step on the autofluorescence and SHG intensity curves: Step 1: the first 30 min in BABB:EtOH (1:1), Step 2: the second 30 min in BABB:EtOH (1:1), Step 3: the first 30 min in 100% BABB, and Step 4: the second 30 min in 100% BABB 100%. Note that the intensities in this comparative study were normalized to the previous step of the clearing process.

# Reference Cited in this Supplementary Material (Appendices)

Chen JS, Pan C, Wu CT, and Liu WK, 1996, Reproducing kernel particle methods for large deformation analysis of non-linear structures, *Comput Methods Appl Mech Eng*, **139** (1–4), 195–227. https://doi.org/10.1016/S0045-7825(96)01083-3.
